# Single nuclei RNA-seq reveals a medium spiny neuron glutamate excitotoxicity signature prior to the onset of neuronal death in an ovine Huntington’s disease model

**DOI:** 10.1093/hmg/ddae087

**Published:** 2024-05-22

**Authors:** Andrew Jiang, Linya You, Renee R Handley, Victoria Hawkins, Suzanne J Reid, Jessie C Jacobsen, Stefano Patassini, Skye R Rudiger, Clive J Mclaughlan, Jennifer M Kelly, Paul J Verma, C Simon Bawden, James F Gusella, Marcy E MacDonald, Henry J Waldvogel, Richard L M Faull, Klaus Lehnert, Russell G Snell

**Affiliations:** Applied Translational Genetics Group, Centre for Brain Research, School of Biological Sciences, The University of Auckland, 3 Symonds Street, Auckland 1010, New Zealand; Department of Human Anatomy & Histoembryology, School of Basic Medical Sciences, Fudan University, 131 Dong'an Road, Shanghai 200032, China; Key Laboratory of Medical Imaging Computing and Computer Assisted Intervention of Shanghai, 130 Dong'an Road, Shanghai 200032, China; Applied Translational Genetics Group, Centre for Brain Research, School of Biological Sciences, The University of Auckland, 3 Symonds Street, Auckland 1010, New Zealand; Applied Translational Genetics Group, Centre for Brain Research, School of Biological Sciences, The University of Auckland, 3 Symonds Street, Auckland 1010, New Zealand; Applied Translational Genetics Group, Centre for Brain Research, School of Biological Sciences, The University of Auckland, 3 Symonds Street, Auckland 1010, New Zealand; Applied Translational Genetics Group, Centre for Brain Research, School of Biological Sciences, The University of Auckland, 3 Symonds Street, Auckland 1010, New Zealand; Applied Translational Genetics Group, Centre for Brain Research, School of Biological Sciences, The University of Auckland, 3 Symonds Street, Auckland 1010, New Zealand; Molecular Biology and Reproductive Technology Laboratories, South Australian Research and Development Institute, 129 Holland Road, Adelaide, SA 5350, Australia; Molecular Biology and Reproductive Technology Laboratories, South Australian Research and Development Institute, 129 Holland Road, Adelaide, SA 5350, Australia; Molecular Biology and Reproductive Technology Laboratories, South Australian Research and Development Institute, 129 Holland Road, Adelaide, SA 5350, Australia; Aquatic and Livestock Sciences, South Australian Research and Development Institute, 129 Holland Road, Adelaide, SA 5350, Australia; Molecular Biology and Reproductive Technology Laboratories, South Australian Research and Development Institute, 129 Holland Road, Adelaide, SA 5350, Australia; Molecular Neurogenetics Unit, Center for Genomic Medicine, Massachusetts General Hospital, 185 Cambridge Street, Boston, MA 02114, United States; Department of Genetics, Harvard Medical School, 25 Shattuck Street, Boston, MA 02115, United States; Molecular Neurogenetics Unit, Center for Genomic Medicine, Massachusetts General Hospital, 185 Cambridge Street, Boston, MA 02114, United States; Department of Neurology, Harvard Medical School, 25 Shattuck Street, Boston, MA 02115, United States; Department of Anatomy and Medical Imaging, Centre for Brain Research, Faculty of Medical and Health Science, The University of Auckland, 85 Park Road, Auckland 1023, New Zealand; Department of Anatomy and Medical Imaging, Centre for Brain Research, Faculty of Medical and Health Science, The University of Auckland, 85 Park Road, Auckland 1023, New Zealand; Applied Translational Genetics Group, Centre for Brain Research, School of Biological Sciences, The University of Auckland, 3 Symonds Street, Auckland 1010, New Zealand; Applied Translational Genetics Group, Centre for Brain Research, School of Biological Sciences, The University of Auckland, 3 Symonds Street, Auckland 1010, New Zealand

**Keywords:** Huntington's disease, single nuclei RNA-seq, prodromal, glutamate excitotoxicity

## Abstract

Huntington’s disease (HD) is a neurodegenerative genetic disorder caused by an expansion in the CAG repeat tract of the huntingtin (*HTT*) gene resulting in behavioural, cognitive, and motor defects. Current knowledge of disease pathogenesis remains incomplete, and no disease course-modifying interventions are in clinical use. We have previously reported the development and characterisation of the *OVT73* transgenic sheep model of HD. The 73 polyglutamine repeat is somatically stable and therefore likely captures a prodromal phase of the disease with an absence of motor symptomatology even at 5-years of age and no detectable striatal cell loss. To better understand the disease-initiating events we have undertaken a single nuclei transcriptome study of the striatum of an extensively studied cohort of 5-year-old *OVT73* HD sheep and age matched wild-type controls. We have identified transcriptional upregulation of genes encoding N-methyl-D-aspartate (NMDA), α-amino-3-hydroxy-5-methyl-4-isoxazolepropionic acid (AMPA) and kainate receptors in medium spiny neurons, the cell type preferentially lost early in HD. Further, we observed an upregulation of astrocytic glutamate uptake transporters and medium spiny neuron GABA_A_ receptors, which may maintain glutamate homeostasis. Taken together, these observations support the glutamate excitotoxicity hypothesis as an early neurodegeneration cascade-initiating process but the threshold of toxicity may be regulated by several protective mechanisms. Addressing this biochemical defect early may prevent neuronal loss and avoid the more complex secondary consequences precipitated by cell death.

## Introduction

Huntington’s disease (HD) is a debilitating neurodegenerative genetic disorder caused by an expanded polyglutamine-encoding CAG repeat in the huntingtin gene (*HTT*) [1]. Individuals with 40 or more CAG codons develop the condition with near complete penetrance [2]. Widespread loss of medium spiny neurons (MSN) in the caudate nucleus and putamen (striatum) contributes to the presenting symptoms that encompass motor, cognitive and behavioural abnormalities. Despite characterisation of the HD defect and advancements in our understanding of disease pathogenesis, no proposed disease-modifying interventions have proved successful in HD clinical trials [3].

Glutamate excitotoxicity as an initiator of neuronal death has been a longstanding hypothesis and research focus in neurodegenerative disorders [4–7]. In HD, it has been proposed that the loss of the MSNs of the striatum is due to elevated synaptic glutamate levels from reduced glutamate clearance and increased glutamate release. This excess synaptic glutamate has been proposed to cause an overactivation of ionotropic glutamate receptors including N-methyl-d-aspartate (NMDA) receptors, α-amino-3-hydroxy-5-methyl-4-isoxazolepropionic acid (AMPA) receptors, and kainate receptors, leading to an influx of calcium ions, chronic membrane depolarisation, oxidative stress, and activation of cell death pathways [4–8]. Evidence for acute ionotropic glutamate receptor-mediated excitotoxicity was shown through the experimental striatal injection of glutamate receptor agonists (glutamic acid, kainic acid, quinolinic acid) in rodents and non-human primates resulting in MSN degeneration and motor dysfunction [9–15]. Further, studies investigating several HD mouse models provided evidence for an association between excessive NMDA receptor signalling and striatal degeneration [16–18].

Our group has previously generated a transgenic ovine model of HD (named *OVT73*) that expresses the full-length human huntingtin (*HTT*) cDNA with a pure CAG repeat length of 69 codons along with a short CAA CAG tract, resulting in a polyglutamine tract of 73 units. The HTT cDNA is under the control of the 1.1 kb segment of the immediate upstream human *HTT* genomic sequence [19]. The transcript expression level from the transgene in the *OVT73* animals is estimated to be about ~10% to 20% of a single normal allele equivalent seen in HdhQ80, YAC128 and rat BACHD HD models [20]. The germline transmission of the 73-unit glutamine coding repeat was stable over three generations [20] and somatic instability was observed to be minimal [21]. The moderate expression of the transgene combined with a somatically stable polyglutamine coding repeat might position this model for the investigation of the initiating stages of HD (prodromal phase). Animals at 6 years of age do not exhibit any striatal cell loss and there are animals that are over 10 years of age not showing any overt symptoms [20, 22–25]. Several molecular and behavioural changes comparable to early phase HD were observed in the *OVT73* line, including the formation of intracellular *HTT* aggregates [20], circadian rhythm abnormalities [24, 25] and changes in brain, plasma and liver metabolites [22, 23, 26, 27]. We have previously reported increased urea levels in the *OVT73* striatum [27] also found on post-mortem throughout HD patients brains including the striatum. The human urea results do not appear to be an end stage cell loss associated phenomena as it is also found in HD post-mortem brains with low grade neuropathology and lower levels of cell loss (Vonsattel grade 0/1) [28]. Increased urea and ammonia levels are known to cause neurological impairment and have been suggested to exacerbate neuronal excitotoxicity [29–33].

To gain a better understanding of the disease mechanism in a prodromal HD model, we have undertaken RNA-seq of single nuclei from the *OVT73* striatum. The tissue utilised for this study was obtained immediately adjacent to that used for our previously reported bulk RNA-seq analysis [27], taken from an extensively investigated cohort of 5-year-old animals [20, 22–25]. Multidimensional data from these animals are also publicly available in the form of a queryable database [34]. Surprisingly, we have detected transcriptomic signatures for a process that indicates the upregulation of glutamate signalling occurring in the MSNs of the *OVT73* striatum. Since our HD sheep do not display striatal cell loss or overt motor symptoms, we propose that the response to glutamate in these animals is at a “steady state” but may be the prelude to excitotoxicity mediated neurodegeneration. These results suggest that there is a treatment window when the biochemical dysfunction could be addressed before the cascade of cell death. These observations position the *OVT73* HD sheep model as a valuable resource testing of therapeutics in the prodromal phase and not at a disease stage confounded by the cell death cascade.

## Results

### Upregulation of genes involved in synaptic transmission in *OVT73* medium spiny neurons

**Figure 1:**
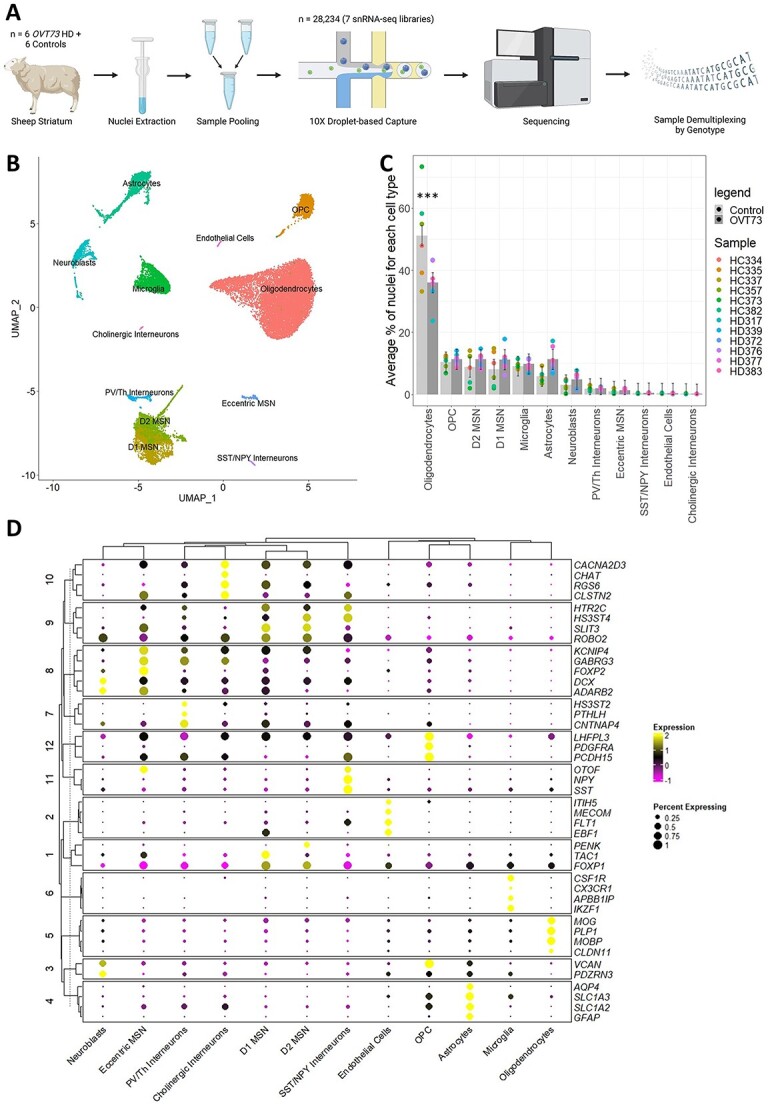
Single nuclei RNA-seq of the *OVT73* sheep striatum. (A) Experimental design. Nuclei were extracted from the striatum of 12 sheep (6 *OVT73* and 6 controls) and pooled to form 7 nuclei suspensions. Single nuclei RNA libraries were generated from the sample multiplexed suspensions and sequenced. Reads were demultiplexed based on the natural genetic variation between pooled samples. Panel image created with BioRender.com. (B) UMAP visualisation of clusters identified and annotated by cell marker gene expression. (C) Proportions of identified cell types across the 12 animals. A significant decrease in oligodendrocytes between *OVT73* and control cases was observed (^*^^*^^*^ANOVA, *P* < 0.001) (D) dot plot of selected cell-type enriched markers for annotated cell types in the sheep striatum.

Following quality control, a total of 28 234 nuclei were recovered from the striatum of 6 *OVT73* sheep and 6 age matched wild-type controls. A total of 13 cell types were identified including 12 507 oligodendrocytes (expressing marker genes *MOG* and *PLP1*), 6093 MSNs (*RGS9*, *PDE10A*), 3024 oligodendrocyte precursor cells (*PCDH15*, *PDGFRA*), 2601 microglia (*CSF1R*, *CX3CR1*), 2181 astrocytes (*AQP4*, *GFAP*), 1068 neuroblasts (*DCX*¸ *ADARB2*), 683 interneurons (*ELAVL2*, *CLSTN2*) and 77 endothelial cells (*FLT*, *MECOM*). Interneurons were subcategorised based on markers defined in Munoz-Manchado *et al*., [35] into 526 PV/Th interneurons (*HS3ST2*, *PTHLH*), 107 SST/NPY interneurons (*SST*, *NPY*) and 50 cholinergic interneurons (*CHAT*). MSNs were subcategorised according to cell markers defined in Saunders *et al*., [36] into 2776 D1 MSNs (*TAC1*, *EBF1*), 2998 D2 MSNs (*DRD2*, *PENK*) and 319 eccentric MSNs (*OTOF*, *FOXP2*). Full cell marker lists are provided in Supplementary File 2, and cell type distributions shown in Fig. 1C. Across all cell types, we observed a significant reduction in the proportion of oligodendrocytes in the *OVT73* derived tissue compared with control cases (ANOVA, *P* = 1.24*×*10^−5^).

**Figure 2:**
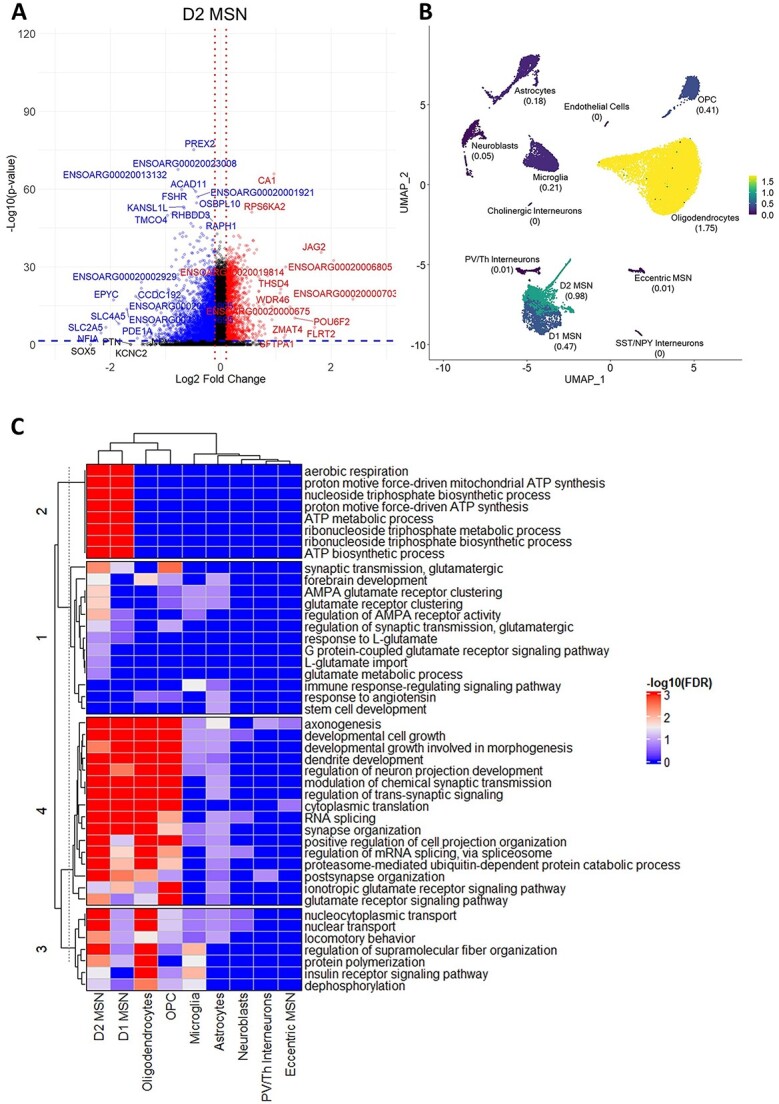
Differentially expressed genes in *OVT73* versus control sheep striatum. (A) Volcano plot of differentially expressed genes (DEGs) identified in D2 medium spiny neurons (*OVT73* D2 MSN vs control D2 MSN). Horizontal blue line shown at *P* = 0.05, vertical red lines shown at log2 fold change of −0.1 and 0.1. (B) Proportion of DEGs over the median number of expressed genes in each cell type. (C) Heatmap of most significant gene ontology terms (ordered by lowest FDR adjusted p-values) in cell types.

To investigate differential gene regulation in the *OVT73* HD striatum, differential expression analyses were conducted between *OVT73* and controls for each cell type separately. The ratio of differentially expressed genes (DEGs) identified to the median number of expressed genes per nucleus was 1.75 in oligodendrocytes (1753 DEGs/1002 median number of expressed genes of oligodendrocyte nuclei), 0.41 in OPCs (742/1812), 0.98 in D2 MSNs (4444/4516), 0.47 in D1 MSNs (2262/4850), 0.01 in eccentric MSNs (45/4337), 0.21 in microglia (217/1049), 0.05 in neuroblasts (47/895), 0.18 in astrocytes (303/1687) and 0.01 in PV/Th interneurons (33/4398) (Fig. 2B). No DEGs were detected in endothelial cells, cholinergic interneurons, and SST/NPY interneurons, likely due to their small population sizes. The statistical power to detect differences in gene expression is dependent on the number of cells, consistent with the highest proportion of DEGs being observed in oligodendrocytes, with the highest number of cells. When comparing the proportion of DEG’s in D1 MSNs, D2 MSNs, OPC, microglia, astrocytes and neuroblasts all represented in the data set by similar nuclei numbers, a greater proportion of DEGs was observed in D2 MSNs. The full list of DEGs is available in Supplementary File 3 and volcano plots for individual cell type DEG analysis are available in Supplementary Fig. 5.

The top enriched Gene Ontology (GO) terms (ordered by FDR adjusted p-values) associated with DEGs included synapse assembly and organisation, GABAergic and glutamatergic synaptic transmission, axonogenesis, neuronal projection development, aerobic respiration and proton motive force-driven mitochondrial ATP synthesis (Fig. 2C). Differential expression in D1 and D2 MSNs were the most significant contributors to the enrichment of the above GO terms. The full list of enriched GO terms is available in Supplementary File 4.

### Co-expressed gene modules involved in synaptic transmission show increased activity in *OVT73* medium spiny neurons and astrocytes

**Figure 3:**
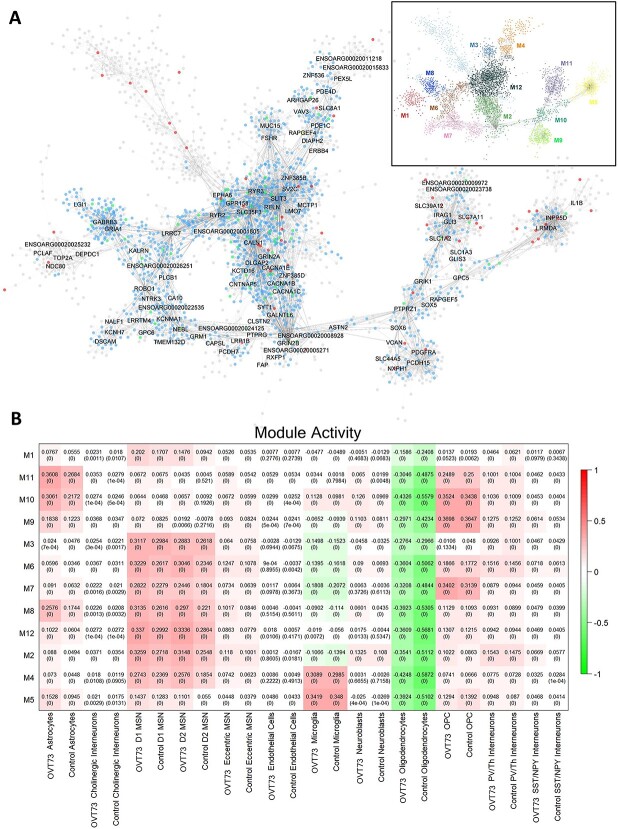
Co-expression gene modules. (A) Co-expression modules generated using the multiscale embedded gene Co-expression network analysis (MEGENA). A total of 12 modules were identified with the structure outlined in the top right. module hub genes are labelled with connected genes represented as dots. Genes differentially expressed between *OVT73* and controls are coloured in blue. Genes with evidence to support an interaction with *HTT* as curated by the HDinHD database [38] were coloured in red. genes that were both differentially expressed and a known *HTT* interactor were coloured in green. (B) Co-expression module activity in *OVT73* and control cell types. Module activity in cell types were determined by computing the module eigengene (first principal component) using normalised expression values of module genes. Eigengene values are shown with p-values of the correlation shown in parentheses in each square. Higher eigengene values indicate higher gene expression of module genes within the cell type.

To identify and investigate sets of genes that were co-expressed in the *OVT73* versus control cell types, we performed co-expression module analysis with MEGENA [37]. We identified several gene modules involved in synaptic transmission that exhibited higher module activity in *OVT73* MSNs and *OVT73* astrocytes compared to controls. A module centred around *DLGAP2*, *GRIN2B*, *CACNA1C*, *CACNA1B*, *CNTNAP5*, *SYT1*, *KCTD16*, and *GRIN2A* genes (Module 2, M2) showed higher module activity in *OVT73* D1 MSNs compared to control D1 MSNs (differential module activity of 0.054; *P* < 0.0005, randomised permutation test with 2000 permutations) and *OVT73* D2 MSNs compared to control D2 MSNs (differential module activity of 0.06, *P* < 0.0005) (Fig. 3B, Supplementary Fig. 7 and Supplementary Fig. 8). The most significant GO terms for module genes included synapse organisation, synapse assembly, chemical synaptic transmission, dicarboxylic acid catabolic process and glutamate metabolic process (Supplementary Fig. 6 and Supplementary File 6). Furthermore, this module was enriched for D1 MSNs DEGs (gene set over-representation analysis, FDR = 0.022) and D2 MSNs DEGs (FDR = 0.0013). Additionally, a module centred around *SLC1A2*, *SLC7A11*, *GLI3*, *IRAG1*, *GFAP* and *SLC39A12* genes (Module 11, M11) was enriched in *OVT73* astrocytes compared to control astrocytes (differential module activity of 0.092, *P* < 0.0005). Most significant GO terms for modules genes included L-glutamate transmembrane transport and amino acid transport. The module showed an enrichment of astrocyte DEGs (FDR = 2.08*×*10^−5^). Full lists of genes in gene modules are available in Supplementary File 5. The full list of enriched GO terms for modules is available in Supplementary File 6.

### Cell–cell signalling is elevated in *OVT73* animals

**Figure 4:**
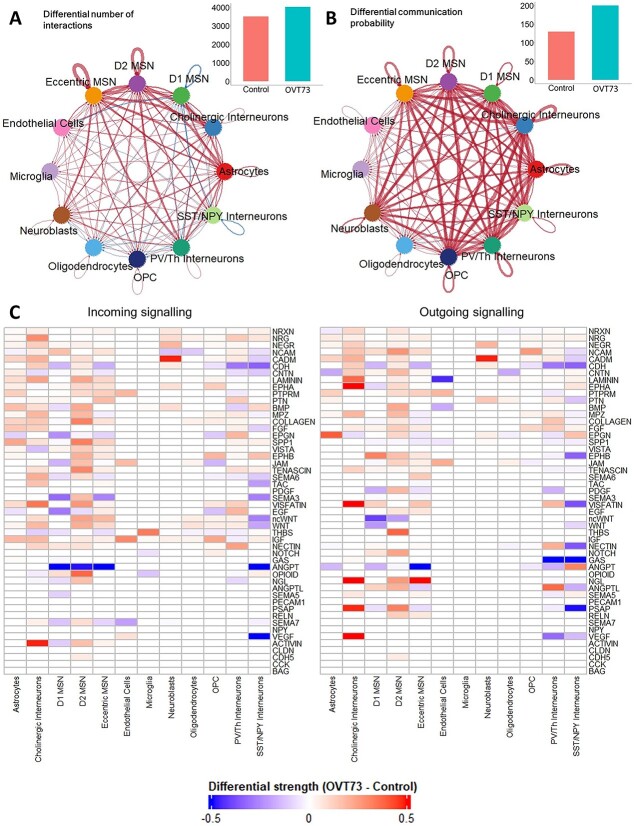
CellChat cell–cell communication networks inferred from expression of ligand-receptor pairs. Circle plot showcasing the (A) differential number of ligand-receptor interactions and (B) differential communication probability between *OVT73* and controls for any two cell types. Red arrows indicate increased number/communication probability in *OVT73*, blue arrows indicate decreased number/communication probability in *OVT73*. Thickness of line indicates greater number/communication probability. The total number of ligand-receptor interactions and total interaction strength for *OVT73* and controls is also shown as bar graphs on the top right. (C) Differential information flow between *OVT73* and control cell types for outgoing (ligand to receptor) and incoming (receptor to ligand) signalling pathways. The information flow for a given signalling pathway is defined as the sum of communication probabilities of all ligand receptor pairs in the pathway. A positive value indicates more communication in *OVT73*.

The differential expression analyses and co-expression analyses both indicated that synaptic signalling and transmission may be increased in the *OVT73* animals. This led us to examine the cell–cell communication networks using CellChat [39]. Cell–cell communication analysis also supported increased synaptic signalling in neuronal and glial cells of the *OVT73* striatum. Comparison of ligand-receptor pair expression from *OVT73* and control revealed a higher total number of ligand-receptor interactions (4030 and 3509 detected ligand-receptor pairs for *OVT73* and control respectively) and a higher sum of all communication probabilities (200 and 130 for *OVT73* and control respectively) across all cell types in the *OVT73* samples. The cell types that showed the greatest increase in the number of incoming (receptor to ligand) and outgoing (ligand to receptor) ligand-receptor interactions included D2 MSNs and astrocytes. The communication probability was greater in the *OVT73* dataset for all neuronal cells (D1, D2, eccentric MSNs and PV/Th, NPY/SST, cholinergic interneurons), OPCs and astrocytes indicating more cell–cell crosstalk in the *OVT73* cell state (Fig. 4A and B, thicker lines indicate higher number of ligand receptor interactions or higher communication probability in *OVT73*). In contrast, no differences in cell–cell signalling were detected in microglia from *OVT73* compared to controls (Supplementary Fig. 9).

Examination of signalling pathways showed greater information flow (defined as the sum of communication probabilities of all ligand receptor pairs in the signalling pathway) in neurexin (NRXN), neuregulin (NRG), protein tyrosine phosphatase receptor M (PTPRM), contactin-1 (CNTN), neuronal growth regulator (NEGR), laminin, cell adhesion molecule (CADM), neural cell adhesion molecule (NCAM), ephrin type receptor A (EPHA) and pleiotrophin (PTN) pathways in *OVT73* D2 MSNs. Interestingly, the greatest increase in communication was observed in myelin protein zero (MPZ), collagen, bone morphogenetic protein (BMP), and fibroblast growth factor (FGF) signalling pathways (Fig. 4C, Supplementary Fig. 10). Individual ligand receptor pairs that showed the greatest increase in communication probability between *OVT73* MSNs and control included *NEGR1*-*NEGR1*, *NRXN1*-*NLGN1*, *CADM1*-*CADM1*, *NRXN3*-*NLGN1*, *CDH2*–*CDH2*, *NRG1*-*ERBB4* and *NRG3*-*ERBB4*. These ligand-receptor pairs have been implicated in various neurodevelopmental processes including myelination [40–43], glutamatergic and GABAergic synapse development [44, 45], neurite outgrowth and general nervous system development [46–53]. Individual ligand receptor pair interactions for astrocytes and MSNs (D1, D2) are shown in Supplementary Fig. 11. Communication probabilities between all ligand receptor pairs across all cell types is available in Supplementary File 7.

### Upregulation of glutamate signalling is attenuated by protective mechanisms

**Figure 5:**
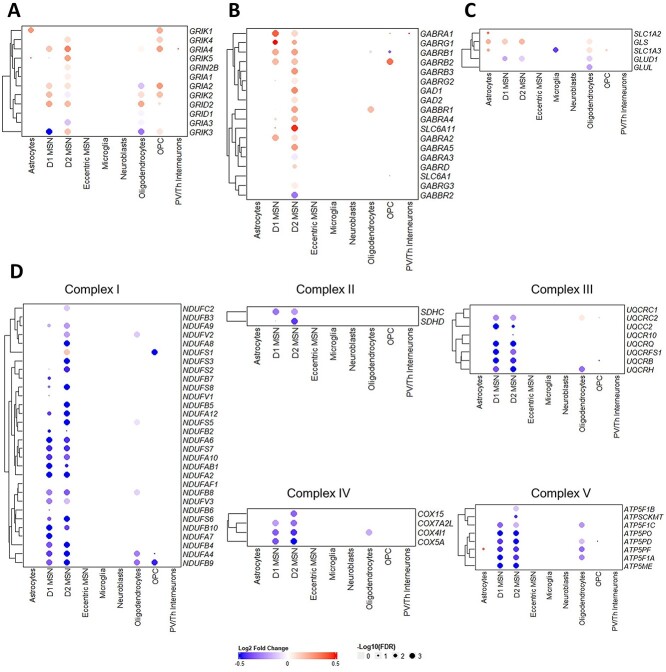
Heatmap of log2 fold change of selected genes between *OVT73* and control cell types implicated in glutamate signalling. Differential gene transcriptional regulation of (A) glutamate receptors (NMDAR, AMPAR, Kainate, delta receptors), (B) GABA_A_ receptors and glutamate to GABA conversion genes, (C) glutamate uptake transporters and glutamine-glutamate cycle genes and (D) genes encoding oxidative phosphorylation complexes.

The glutamate excitotoxicity hypothesis proposes neuronal stress and eventual initiation of cell death pathways arising from elevated levels of synaptic glutamate and excessive signalling through ionotropic glutamate receptors [4–7]. We have identified transcriptional evidence in support of elevated glutamate signalling in the *OVT73* striatum through a widespread transcriptional upregulation of ionotropic glutamate receptors (NMDA, AMPA, kainate and delta receptors) (Fig. 5A). We also observed an upregulation of transcription of the glutamine to glutamate conversion enzyme glutaminase (*GLS*) in *OVT73* D1 and D2 MSNs and *OVT73* astrocytes. Upregulation of *GLS* suggests increased production of glutamate which may be released into the synaptic space, bind glutamate receptors, and trigger a feedback loop to upregulate glutamate receptor expression. Elevated glutamate signalling is commonly observed with oxidative stress signatures represented by reduced activity of the oxidative phosphorylation complexes [54–56]. In keeping with this we have observed a downregulation of several genes encoding oxidative phosphorylation complexes including complex I, II, III, IV and V in *OVT73* D1 and D2 MSNs.

In addition, we observed transcriptional upregulation of several genes likely due to a compensatory response to elevated glutamate levels. The transcription of genes encoding glutamate uptake transporters GLT (*SLC1A2*) and GLAST (*SLC1A3*) were upregulated in *OVT73* astrocytes suggesting a response to remove excess synaptic glutamate (Fig. 5C). We also describe a transcriptional upregulation of genes encoding the gamma-aminobutyric acid A receptor subunits (GABA_A_) including the α, β and γ subunits in *OVT73* D1 and D2 MSNs compared to controls. Additionally, an upregulation of genes encoding GABA transporter GAT4 (*SLC6A11*) and genes encoding the glutamate to GABA conversion enzymes, glutamate decarboxylase (*GAD1* and *GAD2*) was also observed in *OVT73* D2 MSNs (Fig. 5B). Taken together, these observations suggested increased GABA_A_ receptor signalling is likely a result of increased glutamate conversion to GABA.

### Transcription analysis of gene regulatory networks indicate reduced CREB regulon activity in the *OVT73* striatum

**Figure 6:**
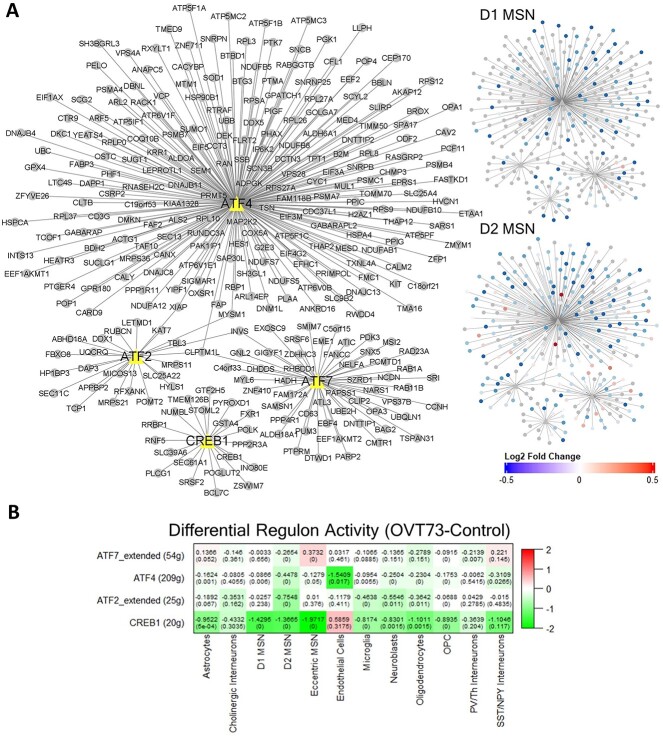
Gene regulatory networks show reduced CREB regulon activity in *OVT73*. (A) CREB-related transcription factor regulated gene modules (regulons) network. The associated transcription factor regulon is shown as triangles with gene members of the regulon connected as dots. The two panels on the right show the log2 fold change of differentially expressed gene members of the regulon between *OVT73* and controls in D1 MSNs and D2 MSNs. (B) Differential regulon activity was computed by subtraction of regulon activity between *OVT73* and controls. A randomised permutation test with 2000 permutations was performed to determine significant differential regulon activity between *OVT73* and control cell types. P-values of the randomised permutation test are shown in the parentheses.

We assessed the transcription factor regulation of highly variable genes in *OVT73* through construction of gene regulatory networks using SCENIC. We focused on the regulon activity of the cAMP-responsive element-binding protein (CREB) family of transcription factors that has been shown to be activated following excessive glutamate signalling [57, 58]. When examining differential regulon activity between *OVT73* and controls, we observed a reduction in regulon activity for *CREB1* in astrocytes (differential module activity of −0.952, *P* = 0.0005, randomised permutation test with 2000 permutations), D1 MSNs (−1.43, *P* < 0.0005) and D2 MSNs (−1.367, *P* < 0.0005) (Fig. 6). *ATF2* regulon activity was reduced between *OVT73* and controls for D2 MSN (−0.755, *P* < 0.0005). *ATF4* regulon activity was reduced between *OVT73* and controls for astrocytes (−0.162, *P* = 0.001), D1 MSNs (−0.087, *P* = 0.006) and D2 MSNs (−0.448, *P* < 0.0005). *ATF7* regulon activity was reduced between *OVT73* and controls for D2 MSNs (−0.265, *P* < 0.0005) (Fig. 6B, Supplementary Fig. 14). The greatest sum of difference in CREB regulon activity between *OVT73* and controls (sum of *CREB1*, *ATF2*, *ATF4*, *ATF7* regulon activity in *OVT73* subtracted by the sum of *CREB1*, *ATF2*, *ATF4*, *ATF7* regulon activity in control) was observed in the D2 MSNs. Interestingly, CREB regulon activity was reduced to a lesser degree in D1 MSNs compared to D2 MSNs. Regulon activity of all identified regulons is available in Supplementary Fig. 12 and Supplementary Fig. 13. Gene members of each regulon are available in Supplementary File 8.

## Discussion

Revealing the HD pathogenic mechanisms prior to striatal cell loss and motor and cognitive deficits is likely to reveal disease modifying pharmaceutical targets. Our group has generated and characterised a transgenic sheep model of HD (named *OVT73*) that displays no striatal cell loss, or overt symptoms but exhibits many of the molecular changes of prodromal HD [20, 22–26]. To gain further insight into cellular changes in a prodromal HD model we undertook a single nuclei transcriptomic study in striatal tissue from 5-year-old *OVT73* animals. Differential gene expression analysis, gene co-expression network analysis and cell–cell signalling analysis indicate alterations in glutamatergic and GABAergic synapses in striatal MSN’s, the most vulnerable cells in HD.

With respect to the glutamatergic synapse, we observed transcriptional upregulation of genes encoding ionotropic glutamate receptors including NMDA, AMPA and kainate receptors in both the D1 and D2 type *OVT73* MSNs. Co-expression analysis also revealed increased transcriptional activity of the gene modules containing the aforementioned glutamate receptor genes in the same cell types. Moreover, cell–cell signalling inferred from ligand-receptor co-expression revealed a disease state increase in signalling of pathways including neurexin, neuroligin, neuregulin, neural cell adhesion molecule and ephrin. These pathways have been implicated in neurite outgrowth, glutamatergic synapse formation and upregulation result in an increase in ionotropic glutamate receptor abundance [44, 59–69]. Previous studies of striatal MSNs from post-mortem human and HD mouse models found positive correlations between ionotropic glutamate receptor mRNA levels and receptor protein abundance. Further, an upregulation of receptor signalling inferred from ligand binding studies and changes in receptor currents were also identified [70–74]. Therefore, it is likely that the observed upregulation of ionotropic glutamate receptor gene transcription in *OVT73* medium spiny neurons indicates increased glutamate signalling.

Overactivation of ionotropic glutamate receptors has been shown to lead to an influx of calcium ions promoting excitotoxic stress, mitochondrial dysfunction, and eventual initiation of cell death [8, 75]. Mitochondrial dysfunction and oxidative stress induced by excitotoxic stress is evident by a decrease in the activity of oxidative phosphorylation (OXPHOS) complexes [76–78]. In support of this, a recent single nuclei RNA-seq study of HD patient striatum and HD mouse model tissue also reports transcriptional downregulation of these OXPHOS complexes in the MSNs [79]. Similarly, in the *OVT73* sheep striatum, we have detected downregulation of transcription from genes encoding OXPHOS complexes including complex I, II, III and IV and V in the *OVT73* D1 and D2 MSNs. This downregulation is occurring in the absence of cell loss in the sheep indicating there must be a compensatory mechanism.

Previous studies have also shown that excess influx of calcium ions due to overactivated ionotropic glutamate receptors can also trigger neuroprotective pathways including phosphorylation of the transcription factor CREB and activation by CREB binding protein [57]. Interestingly, we have observed a downregulation of genes encoding CREB transcription factors (*CREB1*, *ATF2*, *ATF4* and *ATF7*) and a reduction of CREB regulon activity inferred from gene regulatory networks in *OVT73* D1 and D2 MSNs, astrocytes, microglia, neuroblasts and oligodendrocytes. This would suggest that the CREB transcriptional machinery may be dysregulated in *OVT73*. Studies in HD mouse models that have shown that CREB phosphorylation and activation is lost prior to cell death [58, 80–82]. In keeping with the earliest stage selective vulnerability of D2 MSN, the greatest reduction in CREB regulon activity was observed in the *OVT73* D2 MSNs. These results provide further evidence for CREB transcriptional dysregulation in HD and importantly that this precedes the cell death cascade.

In HD, the mutant CAG tract has been shown to expand somatically throughout disease course particularly in the striatum [83–87]. It has been proposed that further repeat expansion in individual cells leads to increased excitotoxic stress in those cells and once a cell specific repeat threshold has been exceeded, cell death processes are activated [88]. The 73-unit polyglutamine repeat coding tract in the *OVT73* line is somatically stable which may explain why there is an absence of striatal neuronal loss. It is possible that this allows us to observe in this model a steady state of compensatory mechanisms activated to prevent damage due to excitotoxic stress. A core mechanism for the clearance of synaptic glutamate is through uptake into astrocytic glutamate transporters (*SLC1A2*, *SLC1A3*) and degradation via the glutamine-glutamate cycle [89, 90]. A reduction in astrocytic glutamate transporters at both RNA and protein level have been reported in HD patients and HD mouse models [74, 91, 92]. Conversely, we have observed an increase in transcription of *SLC1A2* and *SLC1A3* in *OVT73* astrocytes. Given the prodromal nature of the *OVT73* model, we postulate that we are observing a disease timepoint where these compensatory mechanisms remain operational and have not been overwhelmed as per the later stages of the disease. Our sheep dataset exclusively only comprises of transcription, however it has been shown in HD mouse models and HD post mortem tissue that *SLC1A2* and *SLC1A3* mRNA levels were positively correlated with uptake of synaptic glutamate [74, 93, 94]. It is therefore possible that the upregulation of *SLC1A2* and *SLC1A3* in the *OVT73* striatum represent a compensatory response to remove excess synaptic glutamate.

The loss of GABA_A_ receptor mediated neuronal inhibition is considered a key feature of neuronal dysfunction in HD [95, 96]. We have found in the *OVT73* model striatum transcriptional upregulation of genes encoding GABA_A_ receptor subunits α, β, and γ specifically in D1 and D2 MSNs. Other studies have revealed that mRNA levels of GABA_A_ receptors in striatal MSNs (human cases and HD mouse models) correlate with receptor protein abundance and receptor signalling inferred through receptor binding studies and changes in receptor currents [95, 96]. Increased striatal GABA_A_ receptor activity have been postulated to have a neuroprotective effect against excitotoxicity [97, 98] and therefore increased GABA_A_ signalling in *OVT73* may be another mechanism that mitigates glutamate induced stress providing neuronal protection in the *OVT73* striatum.

A limitation of our study is the absence of functional or other analyses to confirm the transcriptional differences identified in the present data. Unfortunately, we did not have further tissue from these animals for further analysis. Future functional characterisation in other cohorts will be critical, not only to confirm results summarised in this study, but also for comprehensive characterisation of the overall *OVT73* sheep model.

The transcriptional alterations are encouraging evidence for an active cell stress response to excess synaptic glutamate. This protective response has prevented or delayed the excitotoxic process in the *OVT73* striatum as there is no cell loss. These observations also suggest that glutamate induced stress may be present long before the initiation of the cell death cascade. Our single nuclei RNA-seq observations corroborates reports of HD mouse models that showcase excitotoxicity begins early on in disease phase before the onset of symptoms [99–101]. Current experimental HD therapeutics targeting glutamate mediated excitotoxicity have included NMDA receptor antagonists (Memantine, Amantadine) that reduce the overactivation of NMDA receptors which have been utilised in clinical trials to limited success [102–105]. One potential explanation for the lack of efficacy of these drugs is that the therapeutic agents were administrated too late in disease progression where the consequences of cell death (neuroinflammatory cytokines, proinflammatory markers and reactive oxygen species) takes over the momentum [106]. In support of this proposition striatal neurons of HD mouse models have shown to gradually become resistant to excitotoxicity modifying agents following disease progression [107–110]. The findings of our single nuclei RNA-seq study indicate that striatal cells can maintain steady state disease resilience and early intervention for instance glutamate depletion may prevent the cell death cascade and lead to better clinical outcomes.

## Methods and materials

### Ovine samples

The cohort maintenance and tissue sampling has been reported in previous publications [22, 27]. Briefly, animals were maintained in a certified, purpose-made research facility at the South Australian Research and Development Institute (SARDI). Animals were kept in large paddocks as a mixture of wild-type controls and transgenic animals and fed *ad libitum*. Post-mortem striatal samples were taken from six 5-year-old *OVT73* (3 females, 3 males) HD sheep as previously described [22, 27] and six age-matched wild-type controls (2 females, 4 males). Tissue collection was performed in accordance with the SARDI/PIRSA Animal Ethics Committee (approval no. 19/02 and 05/12). All experiments performed adhered to the recommendations in the ARRIVE guidelines [111]. In brief, a lethal dose of pentobarbitone sodium solution (Lethabarb, 1 ml/2 kg body weight) was administrated intravenously, the brains were removed from the skulls, dissected into the 5 distinct blocks (Fig. 1) and snap frozen initially on dry ice and then in liquid nitrogen. Samples were wrapped in tinfoil and stored at −80°C until further use.

### Nuclei isolation for single nuclei RNA-seq

Nuclei were extracted from frozen anterior ventral-medial striatal (Supplementary Fig. 1) tissue utilising an adapted protocol from Krishnaswami *et al*., 2016 [112]. Briefly, approximately 50–100 mg of brain tissue was transferred to a dounce homogenizer containing 1 ml homogenization buffer—HB (250 mM sucrose, 25 mM KCl, 5 mM MgCl_2_, 10 mM Tris buffer pH 8.0, 1 μM DTT, 1X protease inhibitor (Sigma), 0.4 U/*μ*l RNaseIn (ThermoFisher Scientific), 0.2 U/*μ*l SuperaseIn (ThermoFisher Scientific), 0.1% Triton X-100). Tissue was homogenised using 5 strokes with the loose dounce pestle A, followed by 10–15 strokes of tight dounce pestle B. The homogenate was filtered through a 40 *μ*m strainer into 5 ml Eppendorf tubes and centrifuged at 1000 rcf (4°C) for 8 min. The supernatant was removed, and the pellet was resuspended in 250 *μ*l of HB. A 50%–29% iodixanol gradient (OptiPrep™ Density Gradient Medium, Sigma) was prepared to allow removal of the myelin layer. 250 *μ*l of 50% iodixanol was added to the nuclei-HB mixture and slowly layered on top of 500 *μ*l of 29% iodixanol in a new Eppendorf tube. The resultant gradient was centrifuged at 13 000 rcf (4°C) for 40 min. The supernatant and myelin were removed, and the purified nuclei pellet was resuspended in a solution containing 1 ml PBS, 1% BSA and 0.2 U/*μ*l RNAse inhibitor. 5 *μ*l of resuspended nuclei was stained with 5 *μ*l trypan blue and the quality and number of nuclei was assessed using the Countess II FL Automated Cell Counter (ThermoFisher Scientific). To reduce the cost per library, nuclei suspensions from different samples were pooled at equal concentrations in groups of 2 (Supplementary Fig. 2) prior to library preparation and demultiplexed as described below.

### Library preparation and single nuclei RNA sequencing

The droplet-based Chromium methodology from 10X Genomics was utilised for the generation of single nuclei libraries. Libraries were prepared according to the Chromium Next GEM Single Cell 3’ Reagent Kits v3.1 as per manufacturer’s instructions. The single nuclei RNA-seq libraries were sequenced on the HiSeq XTen platform. Alignment of reads was performed using the CellRanger v7.0.0 pipeline with STAR v2.7.2a to the sheep Oar_rambouillet_v1.0 reference genome and annotation (Ensembl release 107). Summary statistics for single nuclei RNA-seq libraries are shown in Supplementary Fig. 3.

### Sample demultiplexing

Demultiplexing of pooled nuclei associated barcodes in the CellRanger computed alignments utilized the genetic variation between individual samples. In brief, regions of the transcriptome with high read coverage (> 50) were identified using the featureCounts function from the Subread package [113, 114]. These regions were used as input into Freebayes variant caller to find genomic variants for each barcode. A filtering step was subsequently applied using BCFtools [115] to remove low confidence variant calls (QUAL score < 30). Barcodes were assigned to sample ID by genotype at variant loci with the scSplit algorithm [116]. scSplit employs a hidden state model to assign nuclei associated barcodes from the pooled sample to respective groups based on an expectation-maximisation framework [116]. scSplit input parameters included an expected number of mixed samples of 2 and an estimated doublet percentage of 4% (scSplit -n 2 -d 0.04). scSplit demultiplexing outcome of barcodes is available in Supplementary File 1. The filtered unique molecular identifiers (UMI) feature-barcode matrices generated by CellRanger were split according to the demultiplexed barcodes.

### Quality control and cell clustering

The filtered unique molecular identifiers (UMI) feature-barcode matrices were processed with ICARUS software [117, 118] developed by our group which utilizes the Seurat v4.0 R package [119]. A quality control filter was applied to remove low quality nuclei with gene counts less than 200 or more than 7500. Additionally, nuclei with mitochondrial reads (> 5%) were removed. From the 12 samples, a total of 28 234 high quality nuclei were recovered with an average of 1357 median genes per nucleus (range: 1292–2607), an average of 2672 median unique molecular identifiers (UMIs) per nucleus (range: 1569–3877) and an average percentage of transcripts originating from mitochondrial genes of 1.04% (range: 0.5%–2.02%) (Supplementary Fig. 4).

The read counts were normalised and scaled using the NormalizeData function in Seurat with parameters normalisation.method = LogNormalize and scale.factor = 10 000. Dimensionality reduction was performed on normalised read counts using a set of 3000 top variable genes identified through the FindVariableFeatures function in Seurat. All sheep datasets (6 *OVT73* and 6 Control) were integrated using harmony [120] and cell clustering was performed with the first 30 dimensions, k-nearest neighbour value of 20 and the Louvain community detection algorithm. Cell type annotation was performed by comparison of cluster marker genes identified using the FindAllMarkers function in Seurat with parameters min.pct = 0.25 and logfc.threshold = 0.25 (Supplementary File 2 for list of cell type marker genes) against known striatal cell type markers identified in published single cell RNA-seq datasets [36, 79, 121]. Medium spiny neurons were classified into 3 sub-categories (D1, D2 and Eccentric) using cell markers described in the DropViz atlas [36].

### Differential expression analyses and gene ontology enrichment

Differential expression analyses were conducted for each cell type separately between *OVT73* cases and controls. The mixed model-based MAST statistical test [122] was performed to identify differentially expressed genes (DEGs). Genes with a log2 fold change of > 0.1, p-value adjusted for false discovery rate (FDR < 0.05) and expressed in at least 10% of nuclei from either condition (*OVT73* or control) were considered to be differentially expressed. A Gene Ontology (GO) over-representation analysis was performed on differentially expressed genes using the enrichGO function in the R package ClusterProfiler [123], which identifies enriched GO terms using Fisher’s exact test. A list of DEGs was used as input into enrichGO. GO terms were extracted from the NCBI annotation of *Ovis aries*, retrieved with record AH107722 with the R package AnnotationHub [124].

### Gene co-expression analysis

Co-expression analysis was performed for the 3000 most variable genes (identified by the FindVariableFeatures function in Seurat) across all *OVT73* and control samples. MEGENA (v1.5) was used to extract significant gene interactions and construct co-expression gene modules [37]. Default parameters detailed in the MEGENA vignette were used, including the use of Pearson’s correlations, FDR threshold of 0.05, module and hub significance *P*-values of 0.05 and a minimum module size of 10 genes. Modules were visualised graphically using Cystoscape v3.9.1 [125]. Module activity in cell types were determined by computing the module eigengene (first principal component) using normalised expression values of module genes. Higher eigengene values indicate higher gene expression of module genes within the cell type. The WGCNA package was used to compute module eigengenes with the moduleEigengenes function. Differential module activity between *OVT73* and control cell types was computed as a subtraction of module eigengenes of the two conditions. A randomised permutation test was used with 2000 permutations to identify significant differential module activity between *OVT73* and control cell types.

Additionally, Gene Ontology analysis of module genes was performed using the moduleGO function in the DGCA R package with a p-value threshold of 0.05. Gene sets in each module were examined for its enrichment of *OVT73* versus control differentially expressed genes. Gene set enrichment analysis was performed using the enricher function in the clusterProfiler v4.6.0 R package [123]. A minimum and maximum gene set size of 10 and 500 was used with adjusted p-value FDR threshold of 0.05.

### Gene regulatory network analysis

Examination of gene regulatory networks for cell types across *OVT73* and control samples was conducted using R SCENIC v1.2.0 [126]. SCENIC performs cis-regulatory transcription factor binding motif analysis on a set of co-expressed transcription factors and genes. Transcription factor genes were defined from the curated list by Lambert *et al*., [127]. Given, a public sheep transcription factor motif database was not available, a custom transcription factor motif database for sheep was made for this study. The motif database was generated as follows, the 500 bp sequence upstream and 100 bp sequence downstream of the transcriptional start site (TSS) for each gene was extracted from the oar_rambouillet v1.0 reference genome (ensemble version 107). Additionally, the 10 kb sequence upstream and downstream of the TSS were also extracted. The files are available on zenodo (https://doi.org/10.5281/zenodo.8057929) under oar_rambouillet_v1.0_500bp_up_100bp_down.fa and oar_rambouillet_v1.0_10kbp_up_10kbp_down.fa. These regions were assessed for transcription factor binding motifs based on the 2022 SCENIC+ motif collection (https://resources.aertslab.org/cistarget/motif2tf/) using the create_cistarget_motif_databases.py function of the create_cisTarget_databases package (https://github.com/aertslab/create_cisTarget_databases). Code used to generate database is shared under https://doi.org/10.5281/zenodo.8057929.

The 3000 most variable genes (identified by the FindVariableFeatures function in Seurat) across all *OVT73* and control samples were used as inputs into SCENIC. Co-expression modules were constructed using GENIE3 [126, 128] and transcription factor motifs were scored using RcisTarget v1.18.2. Transcription factor-regulated gene modules (regulons) with 10 or more genes were considered. The activity of each regulon in *OVT73* and control cell types (regulon activity) were scored using the AUCell algorithm which computes the enrichment of regulons as an area under the recovery curve across a ranking of all genes in a cell based on their normalised expression values. A high regulon activity indicates genes within the regulon are positively regulated by the transcription factor. Regulons were visualised graphically using Cystoscape v3.9.1 [125]. Differential regulon activity between *OVT73* and control cell types was computed as a subtraction of regulon activity of the two conditions. A randomised permutation test was used with 2000 permutations to identify significant differential regulon activity between *OVT73* and control cell types. Additionally, a regulon specificity score (RSS) was determined which employs the Jensen-Shannon divergence metric to assess cell-type specificity of regulon activity [126]. Regulons in cell types with a specificity score of 1 indicates exclusive expression of the regulon in that one cell type, while a specificity score of 0 indicates the regulon is evenly expressed across all cell types.

### Cell–cell communication analysis

The inference of cell signalling crosstalk through the transcription levels of ligand receptor pairs was examined using CellChat. CellChat incorporates a comprehensive database of ligand receptor interactions, soluble agonists/antagonists and stimulatory/inhibitory membrane bound co-receptors to infer cell–cell communications from single cell RNA-seq data based on mass action models, social network analysis tools and pattern recognition methods [39]. In brief, intercellular communications were inferred through 3 steps; (1) identification of differentially expressed ligands and receptors genes for each cell type using the Wilcoxon rank sum test with a p-value threshold of 0.05. (2) A communication probability (interaction strength) is computed by modelling ligand-receptor interactions using the law of mass action on average expression values of a ligand in one cell group and a receptor of another cell group. This calculation accounts for the number of cells within each cell group. (3) Significant interactions are identified using a permutation test that randomly permutes cell group labels and recomputes the interaction probability.

A comparison of cell–cell communication in *OVT73* versus control cell types was performed using default parameters detailed in the CellChat v1.6.1 vignettes. Ligand-receptor interactions were extracted from the human CellChat database. Interactions involving 50 or less nuclei were filtered out using the filterCommunication function.

## Supplementary Material

Supplementary_F1_ddae087

Supplementary_F2_ddae087

Supplementary_F3_ddae087

Supplementary_F4_ddae087

Supplementary_F5_ddae087

Supplementary_F6_ddae087

Supplementary_F7_ddae087

Supplementary_F8_ddae087

Supplementary_File_legends_ddae087

Supplmentary_Figures_HMG_ddae087

## Data Availability

The data have been deposited in NCBI’s Gene Expression Omnibus (GEO) [129] and are accessible through accession number GSE229839 (https://www.ncbi.nlm.nih.gov/geo/query/acc.cgi?acc= GSE229839). The code used in this study are deposited on zenodo available at https://doi.org/10.5281/zenodo.8057929.
