# Supplementary material for: Single nuclei RNA-seq reveals a medium spiny neuron glutamate excitotoxicity signature prior to the onset of neuronal death in an ovine Huntington’s disease model
Source: Hum Mol Genet. 2024 May 22;33(17):1524–39. doi: 10.1093/hmg/ddae087 (PMC11336116; doi:10.1093/hmg/ddae087)
Supplement: Supplementary_File_legends_ddae087 [file supplementary_file_legends_ddae087.docx]

Supplementary File 1 Nuclei associated barcodes from single nuclei RNA libraries.

Supplementary File 2 Cell type marker genes.

Supplementary File 3 Differentially expressed genes between OVT73 and controls in each cell type.

Supplementary File 4 Gene Ontology enrichment of differentially expressed genes.

Supplementary File 5 Gene members of MEGENA co-expression modules

Supplementary File 6 Gene Ontology enrichment of MEGENA co-expression module genes.

Supplementary File 7 Communication probabilities between all ligand-receptor pairs across all cell types in OVT73 and control

Supplementary File 8 Gene members of regulons
