# Supplementary material for: Single nuclei RNA-seq reveals a medium spiny neuron glutamate excitotoxicity signature prior to the onset of neuronal death in an ovine Huntington’s disease model
Source: Hum Mol Genet. 2024 May 22;33(17):1524–39. doi: 10.1093/hmg/ddae087 (PMC11336116; doi:10.1093/hmg/ddae087)
Supplement: Supplmentary_Figures_HMG_ddae087 [file supplmentary_figures_hmg_ddae087.docx]

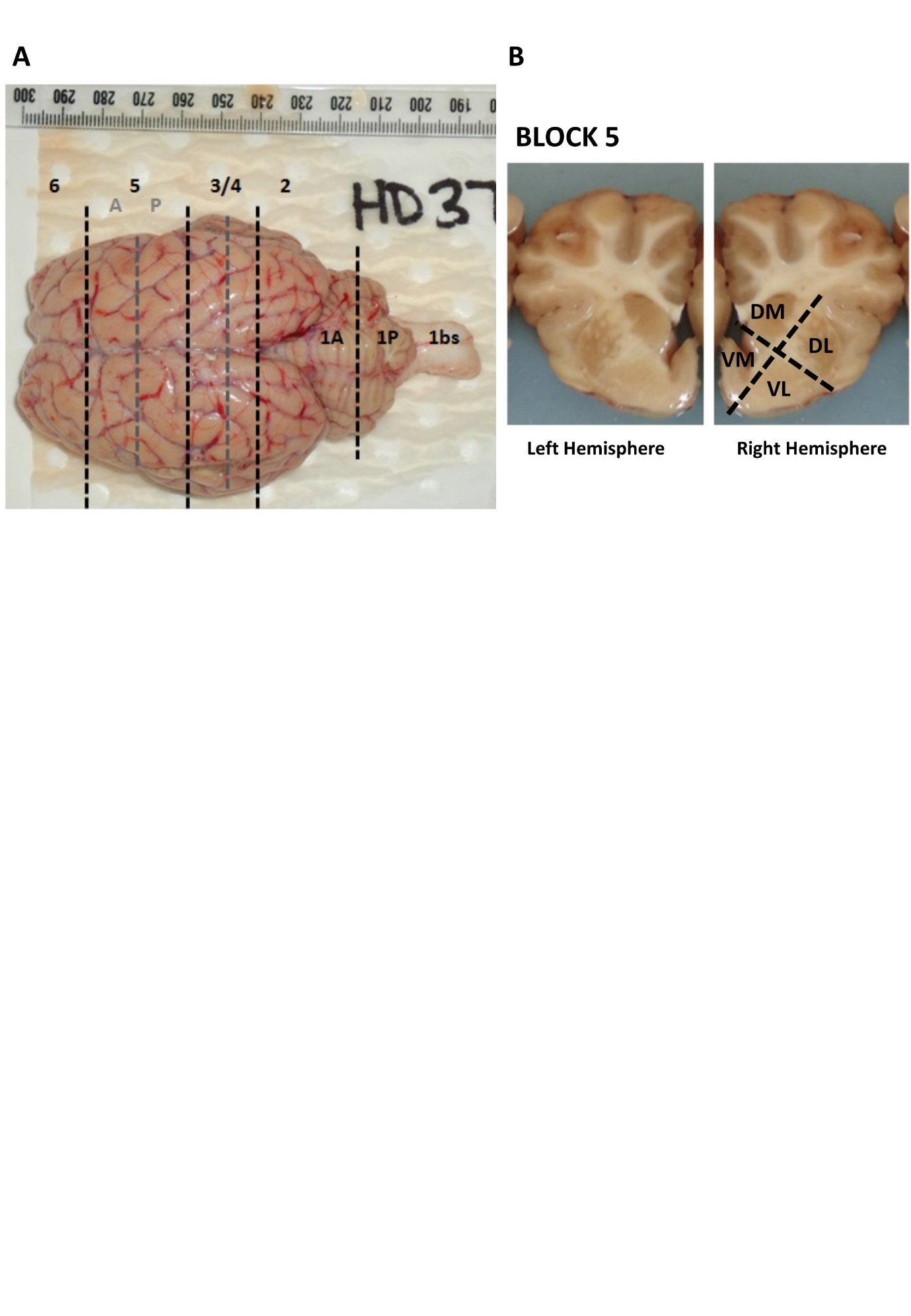
**Supplementary Figure 1** **Overview of brain sectioning procedure during sample collection.** (A) Superior view of a freshly extracted sheep brain showing positions of coronal sectioning. Black dashed lines indicate the initial blocks made from the whole brain. Grey dashed lines indicate sections made from the initial blocks. Block 5 was further subdivided into 5A (anterior) and 5P (posterior). (B) Left and right hemisphere of block 5 sectioning. The striatum was identified within block 5 and further subdivided into 4 subsamples, denoted as dorsal-lateral (DL), dorsal-medial (DM), ventral-medial (VM) and ventral-lateral (VL). The ventral medial sample from the anterior striatum (5A) of the left hemisphere was utilised for the generation of single nuclei transcriptomes.

Supplementary Figure 2 Multiplexed single nuclei RNA-seq libraries

| 10X Library | Samples | Case-control group |
| --- | --- | --- |
| 1 | HC373, HC337 | Control |
| 2 | HC382, HC335 | Control |
| 3 | HD372, HD317 | *OVT73* |
| 4 | HC357 | Control |
| 5 | HD377, HD339 | *OVT73* |
| 6 | HC334 | Control |
| 7 | HD376, HD383 | *OVT73* |

| **10X library** | **1** | **2** | **3** | **4** | **5** | **6** | **7** |
| --- | --- | --- | --- | --- | --- | --- | --- |
| **Estimated #Nuclei** | 4,274 | 4,798 | 4,446 | 4,114 | 5,548 | 3,277 | 8,815 |
| **% Sequencing Saturation^1^** | 55.2 | 49.8 | 57.0 | 45.2 | 36.4 | 50.0 | 47.0 |
| **Total Reads** | 269,984,512 | 247,433,323 | 257,211,090 | 145,901,637 | 241,316,556 | 163,670,979 | 377,815,731 |
| **Total Genes detected** | 20,866 | 20,675 | 20,554 | 20,094 | 20,784 | 20,240 | 21,520 |
| **Median UMI/Barcode** | 1,902 | 1,766 | 2,162 | 2,064 | 2,386 | 2,212 | 2,612 |
| **Mean Reads/Barcode** | 63,169 | 51,570 | 57,852 | 35,465 | 43,496 | 49,945 | 42,861 |
| **Median Genes/Barcode** | 1,115 | 1,032 | 1,188 | 1,090 | 1,202 | 1,192 | 1,331 |
| **% Fraction Reads in Barcode** | 77.8 | 75.1 | 78.8 | 82.6 | 84.6 | 85.9 | 90.3 |
| **% Reads Mapped to Genome** | 98.9 | 98.7 | 98.9 | 98.9 | 98.6 | 98.9 | 96.0 |
| **% Reads Mapped to Transcriptome** | 25.4 | 30.6 | 32.8 | 32.5 | 32.6 | 33.1 | 40.0 |
| **% Reads Mapped to Exonic Regions** | 7.6 | 8.9 | 8.6 | 7.2 | 6.5 | 7.3 | 6.5 |
| **% Reads Mapped to Intronic Regions** | 57.8 | 55.1 | 55.7 | 56.9 | 57.3 | 56.3 | 53.4 |
| **% Reads Mapped to Intergenic Regions** | 27.6 | 28.4 | 28.9 | 29.8 | 29.5 | 30.4 | 30.2 |
| **% Reads Mapped Antisense to Gene** | 39.8 | 33.3 | 31.4 | 31.6 | 31.1 | 30.4 | 19.8 |
| **% Q30 Bases in Barcode** | 97.2 | 97.3 | 96.9 | 97.1 | 96.6 | 97.0 | 97.0 |
| **% Q30 Bases in RNA Read** | 91.6 | 91.7 | 90.6 | 91.4 | 91.0 | 91.5 | 91.3 |
| **% Q30 Bases in UMI** | 97.3 | 97.4 | 96.9 | 97.1 | 96.6 | 97.1 | 96.9 |

Supplementary Figure 3 Summary statistics for multiplexed single nuclei RNA libraries

^1^Sequencing saturation defined as the fraction of reads originating from an already-observed UMI


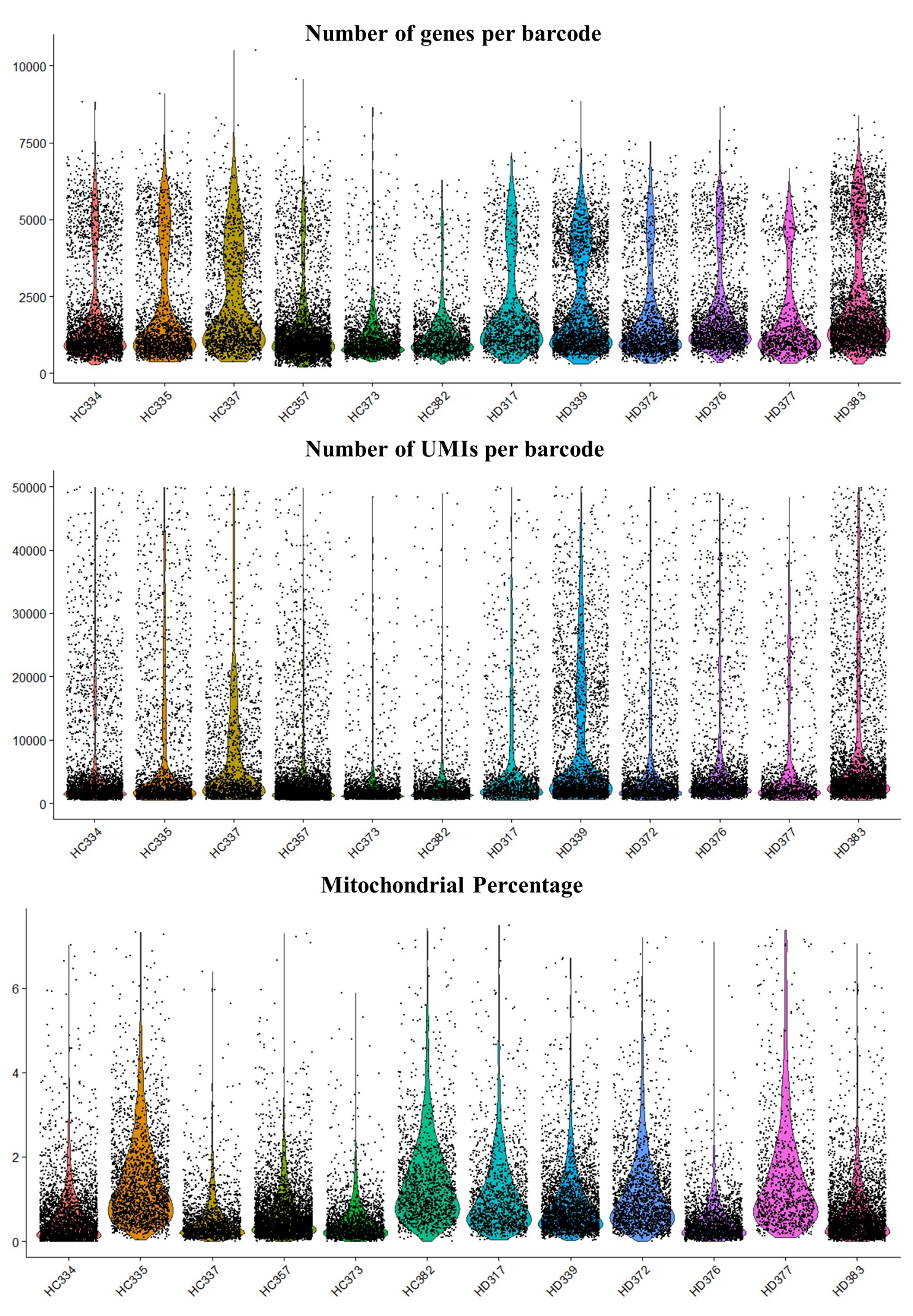
**Supplementary Figure 4 Quality control metrics for single nuclei RNA libraries.**


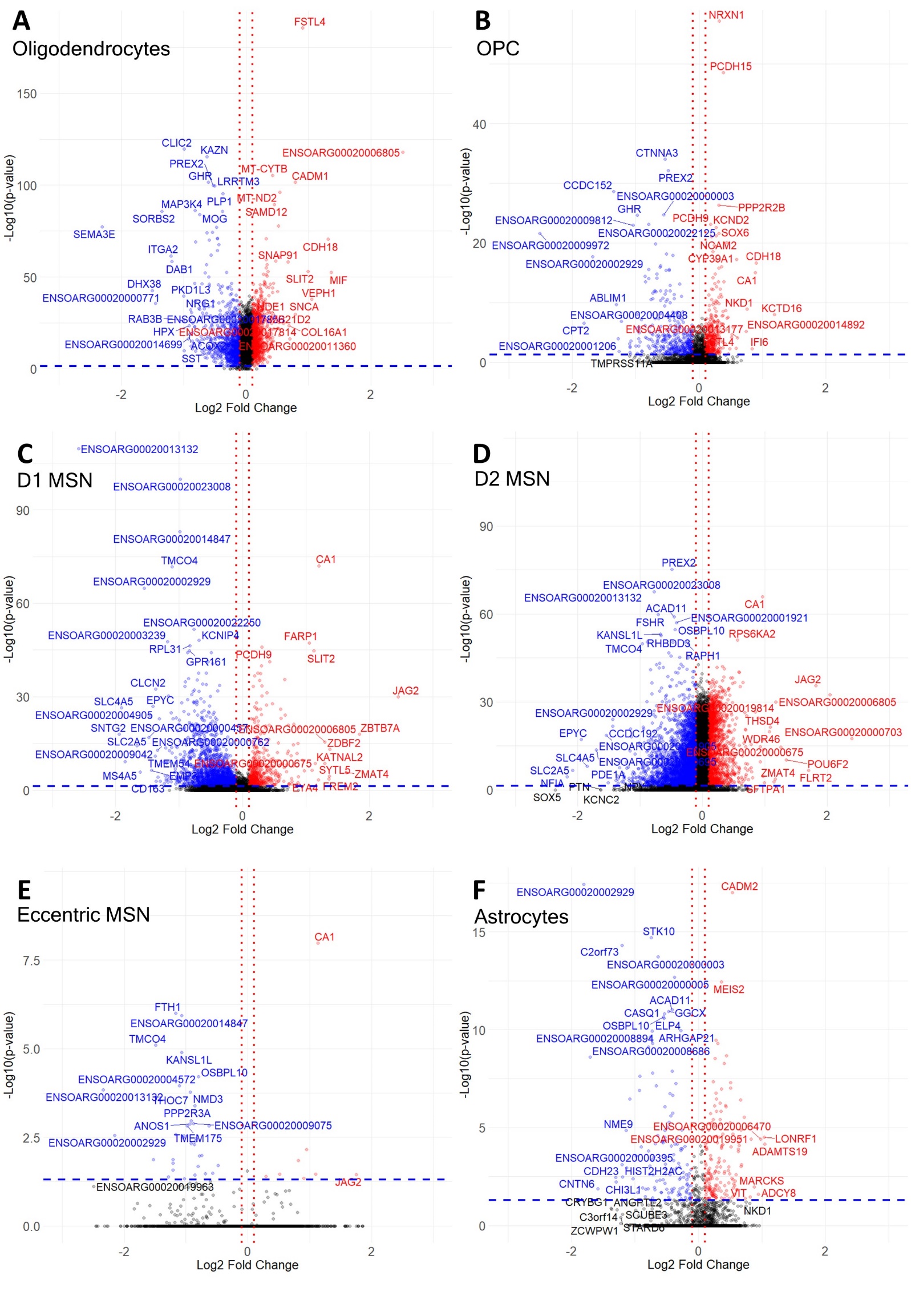


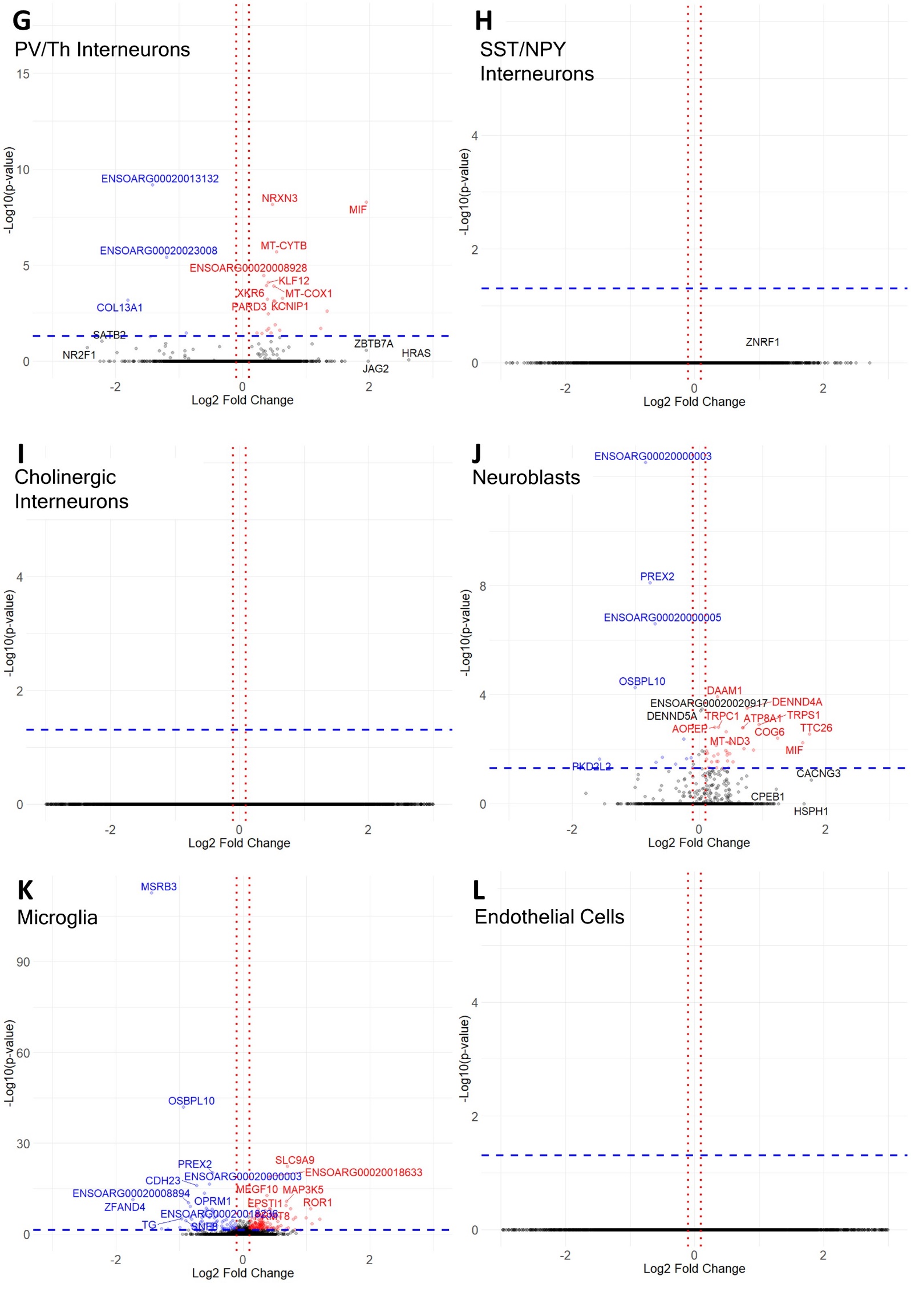


**Supplementary Figure 5 Volcano plot of differentially expressed genes (DEGs) between *OVT73* and control for each cell type identified in the sheep striatum.** Horizontal blue line shown at p=0.05, vertical red lines shown at log2 fold change of -0.1 and 0.1.

**
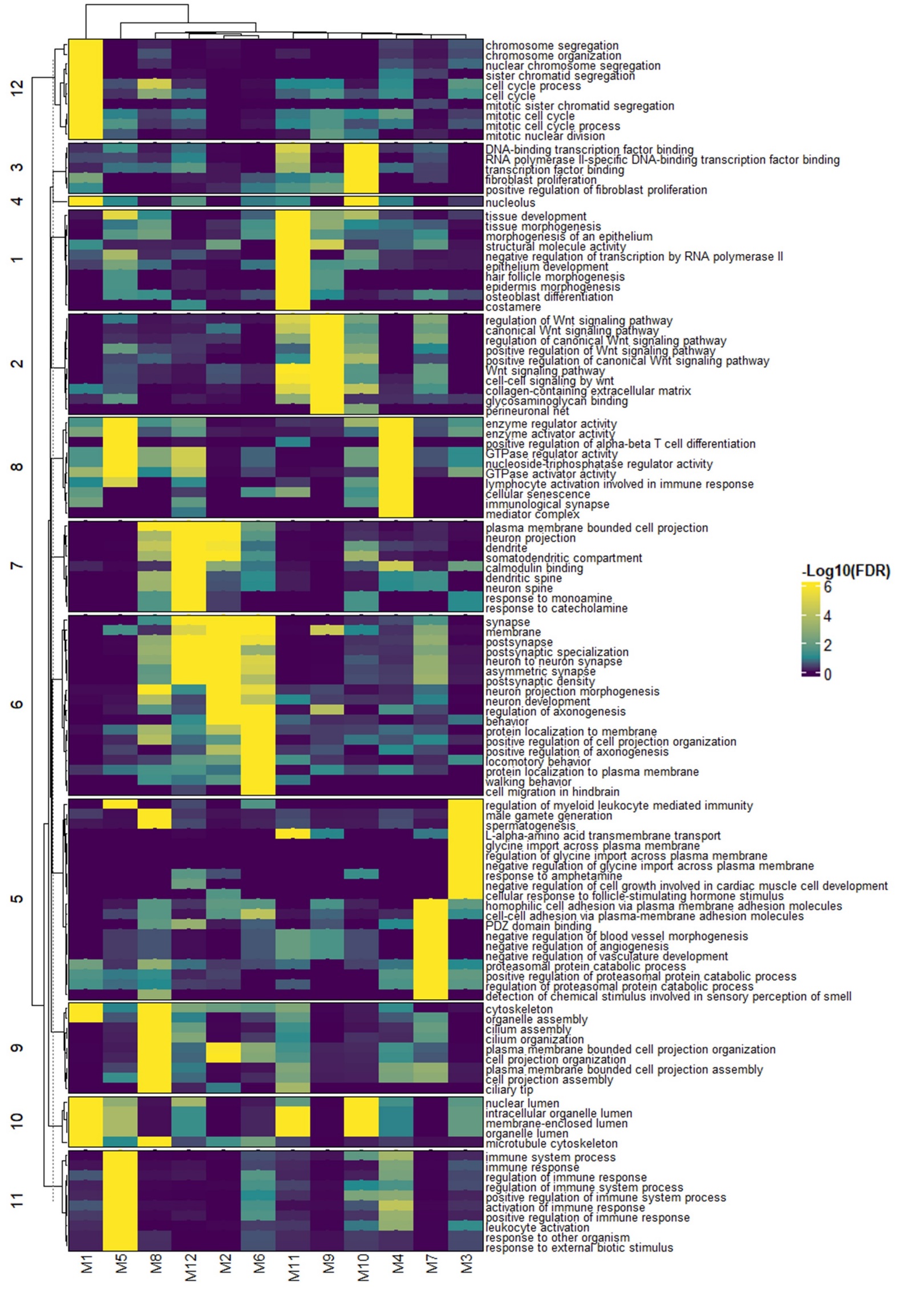
Supplementary Figure 6 Clustered heatmap of gene ontology terms for gene modules.** The 10 most significant gene ontology terms ordered by FDR adjusted p-values for each gene module are shown.


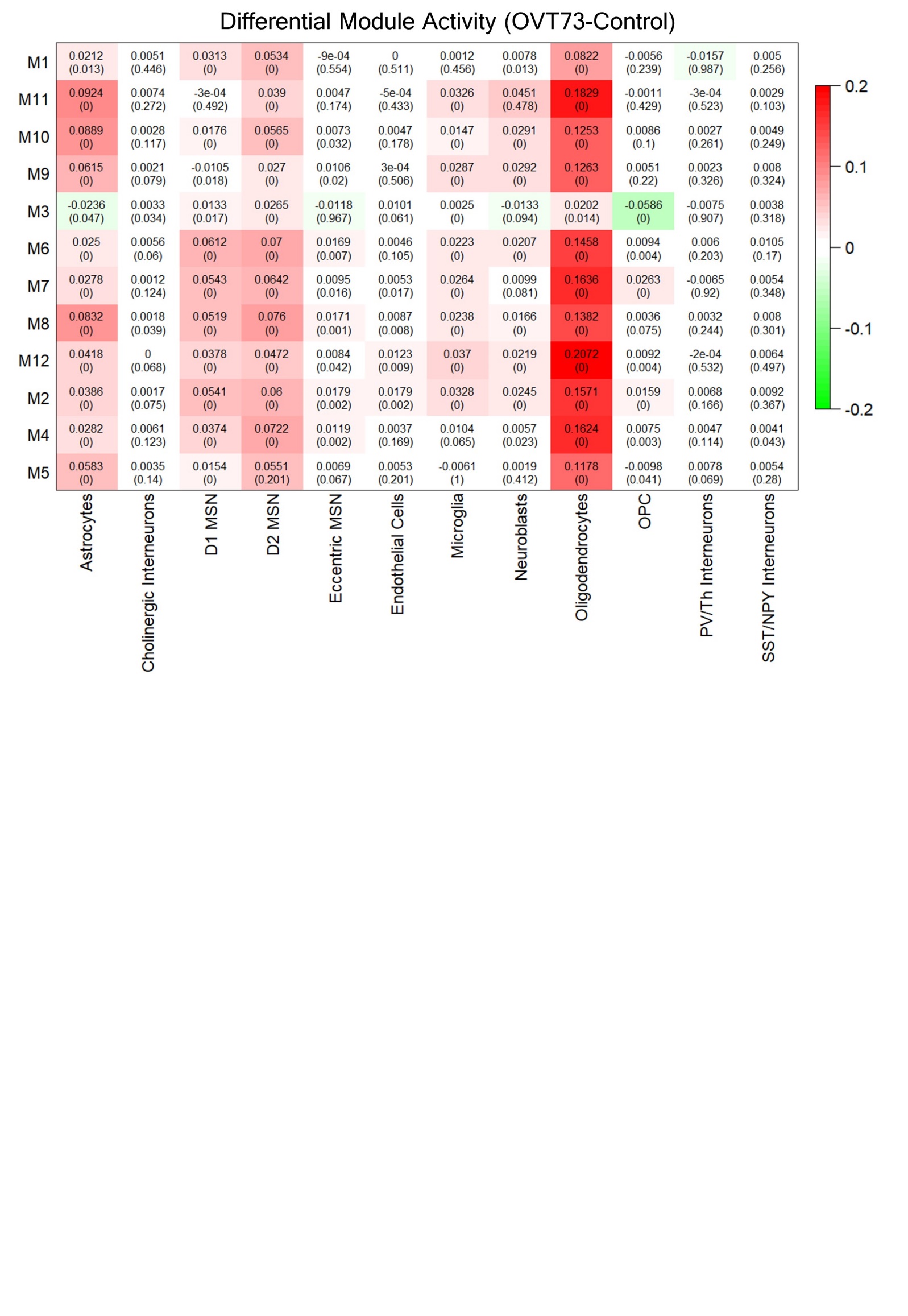


Supplementary Figure 7 Differential co-expression module activity between *OVT73* and control cell types. Module activity in cell types were determined by computing the module eigengene (first principal component) using normalised expression values of module genes. Differential module activity was computed by subtraction of module eigengene values in *OVT73* and control cell types. A randomised permutation test with 2000 permutations was performed to determine significant differential module activity between *OVT73* and control cell types. P-values of the randomised permutation test are shown in the parentheses.

**
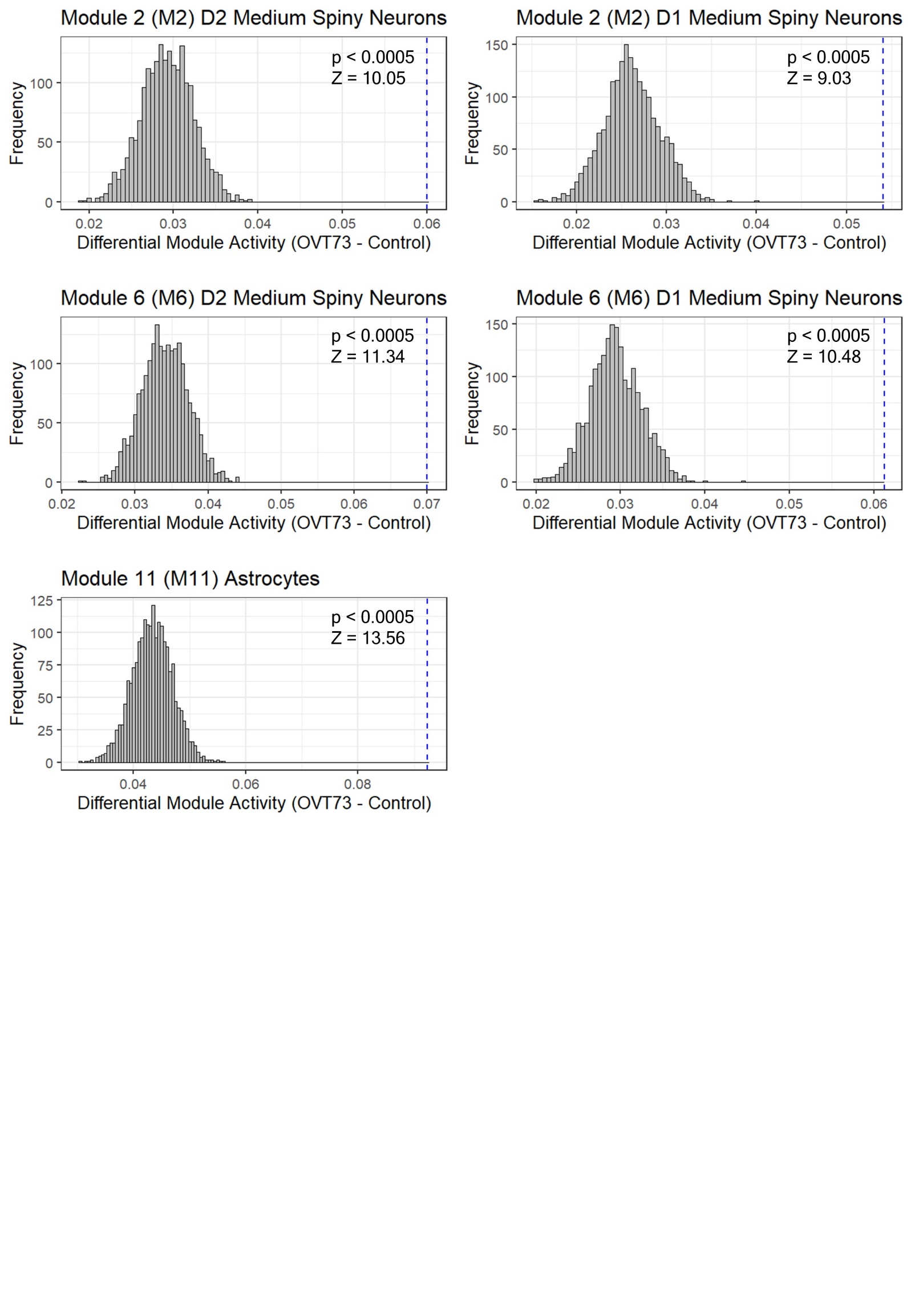
Supplementary Figure 8 Distribution of expected differential module activity scores between *OVT73* and control when genotype labels are randomly assigned.** Randomised permutation tests was performed with 2,000 permutations. Blue vertical line indicates actual differential module activity score.


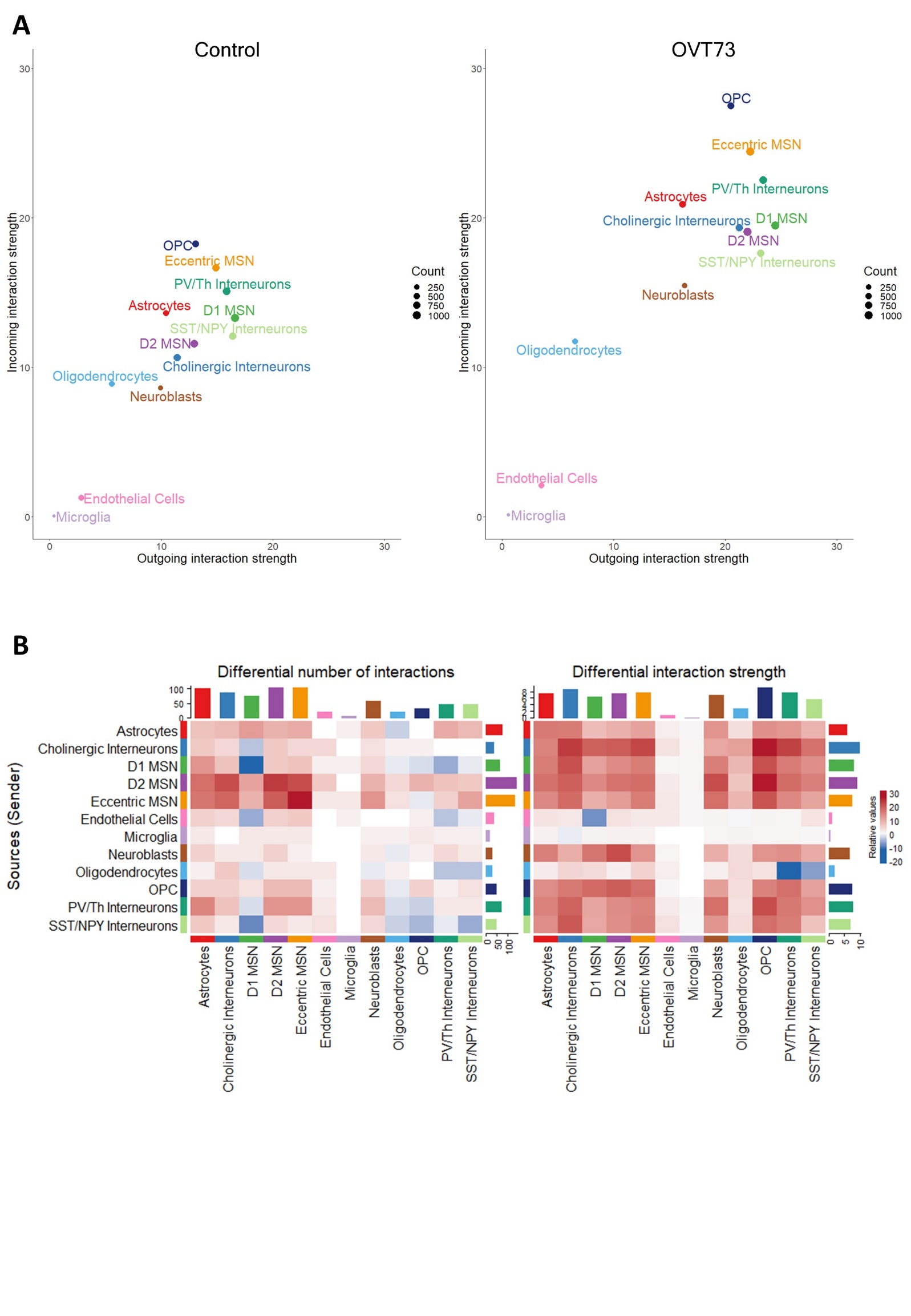


**Supplementary Figure 9 Further visualisations of CellChat cell-cell communication networks.** (A) Comparison of the cell types that exhibit different degrees of incoming interaction signalling (from other cell types) and outgoing interactions signalling (to other cell types) in *OVT73* and control. An overall decrease in signalling was observed for the control dataset compared to *OVT73*. (B) Heatmap of differential number of interactions or differential interaction strength (communication probability) between any two cell types. Red represents increased signalling in the *OVT73* cell types compared to control, blue represents decreased signalling in the *OVT73* cell types compared to control. An overall increase in the number of interactions and interaction strength was observed for the *OVT73* cell types compared to control. The top-coloured bar plot represents the sum of the columns of values displayed in the heatmap (incoming signalling). The right bar plot represents the sum of row of values (outgoing signalling).


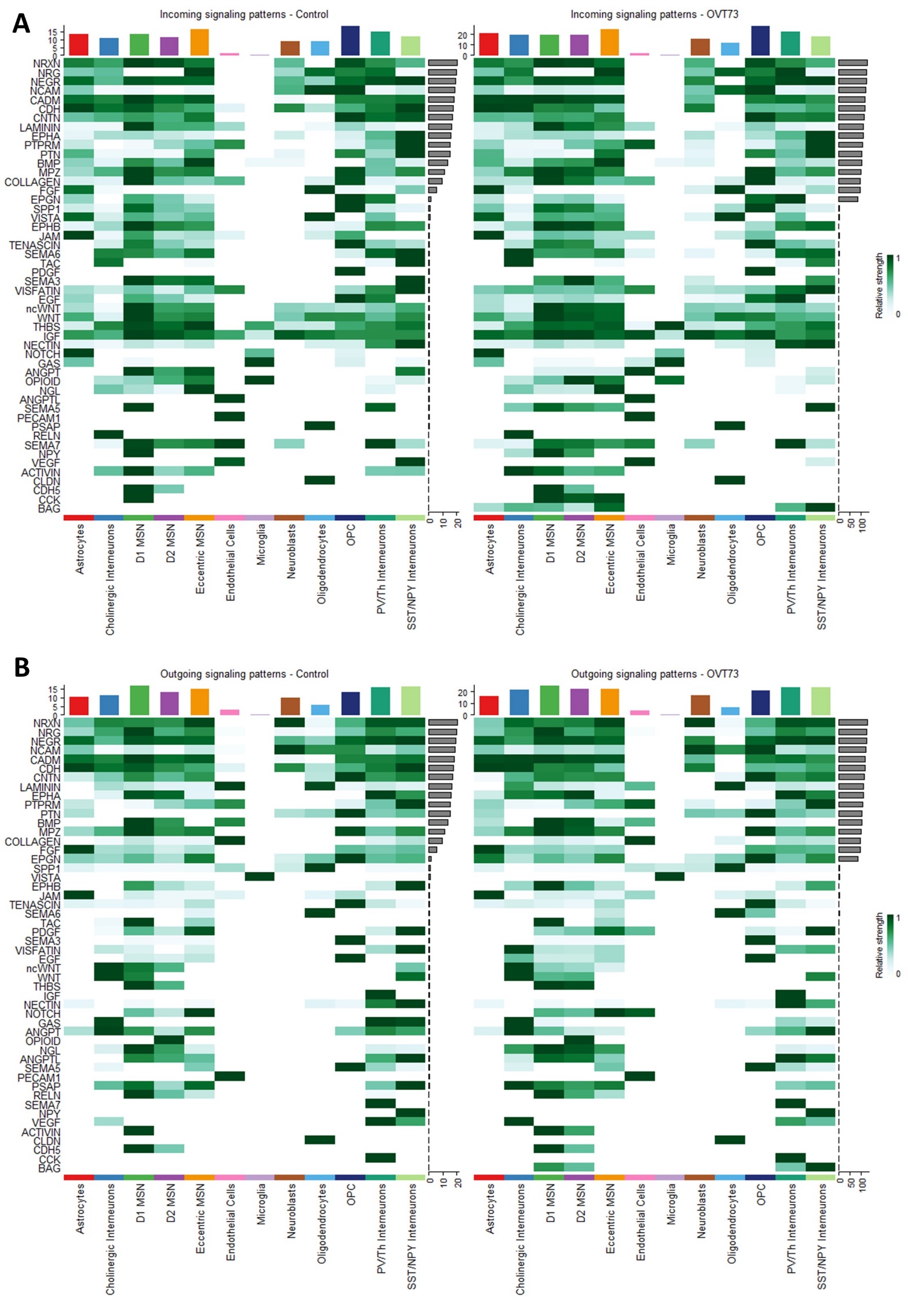
**Supplementary Figure 10 Information flow from outgoing and incoming signalling pathways.** Information flow is defined as the sum of communication probabilities (strength) of all ligand receptor pairs in the signalling pathway.


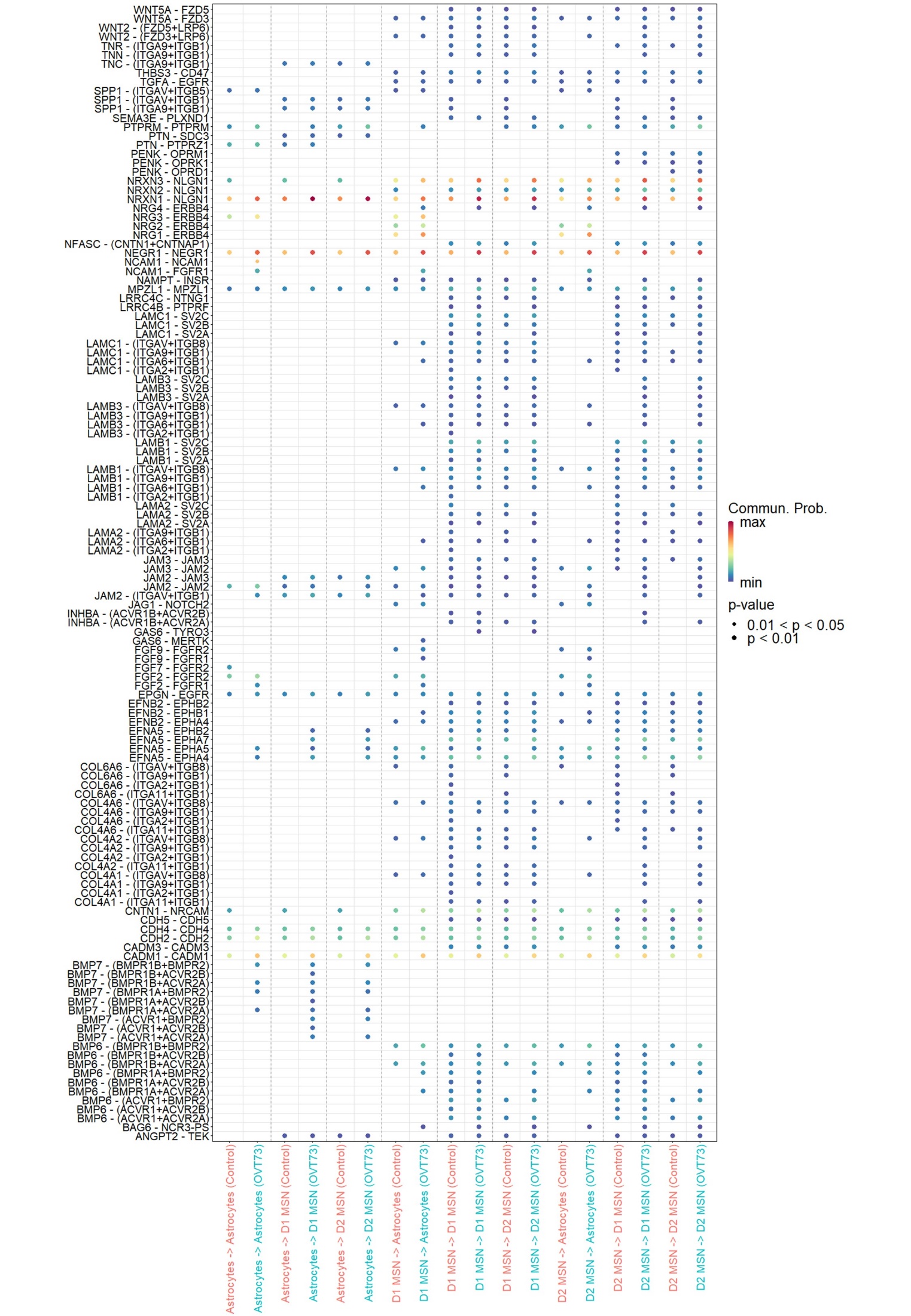
**Supplementary Figure 11 Dot plot of ligand-receptor interactions for *OVT73* and control astrocytes and medium spiny neurons (D1, D2).** Color of the dot represents the communication probability; size of the dot represents the p value. Cell-cell communication probabilities were inferred from ligand receptor expression in single nuclei RNA-seq data using CellChat.

**
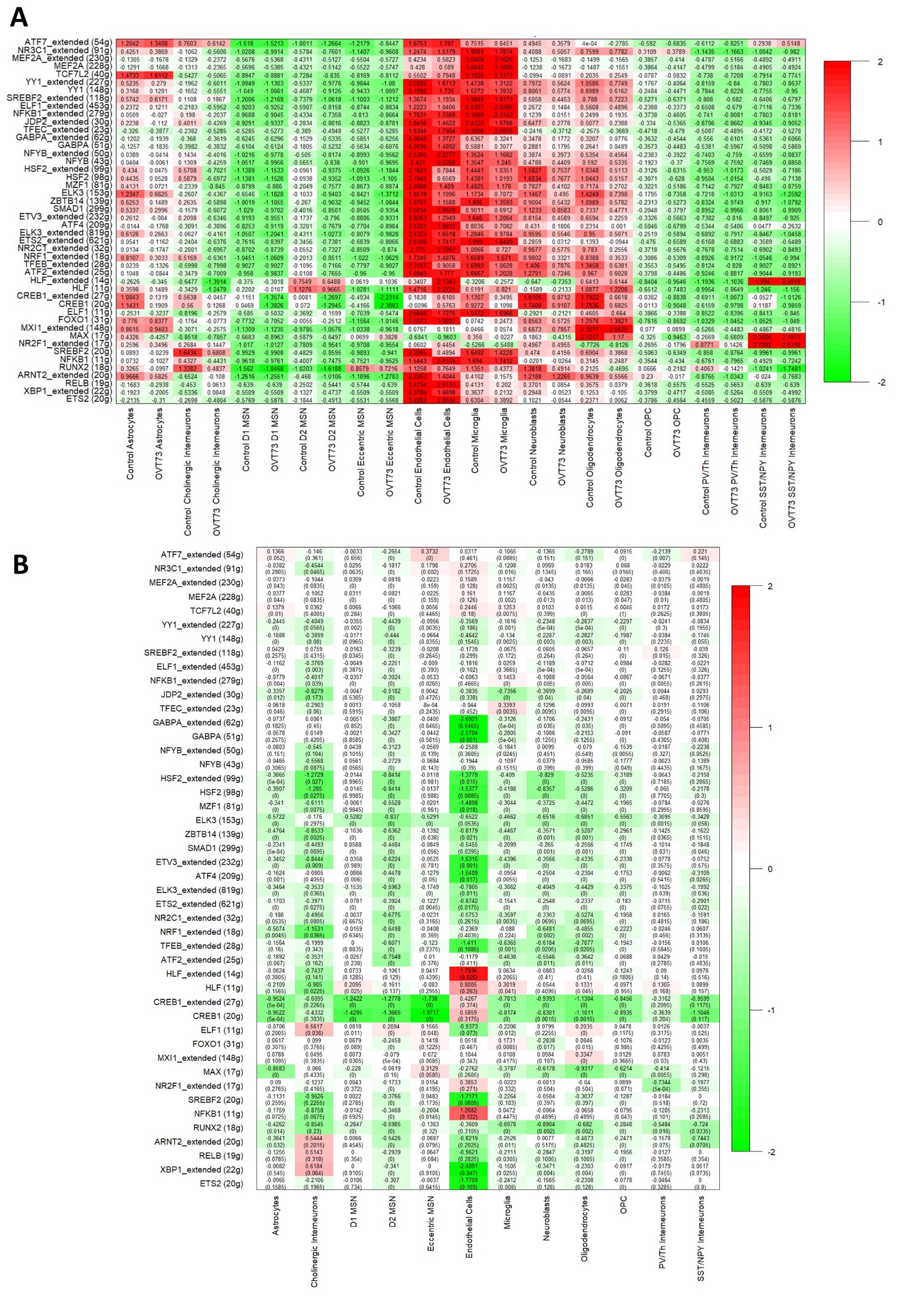
Supplementary Figure 12 Heatmap of gene regulatory network (regulon) activity in OVT73 and control cell types.** (A) Regulon activity was computed based on transcription factor regulated gene modules using OVT73 differentially expressed genes as input. A high regulon activity indicates genes within the regulon are positively regulated by the transcription factor. (B) Differential regulon activity was computed by subtraction of regulon activity in OVT73 and control cell types. A randomised permutation test with 2000 permutations was performed to determine significant differential regulon activity between OVT73 and control cell types. P-values of the randomised permutation test are shown in the parentheses.


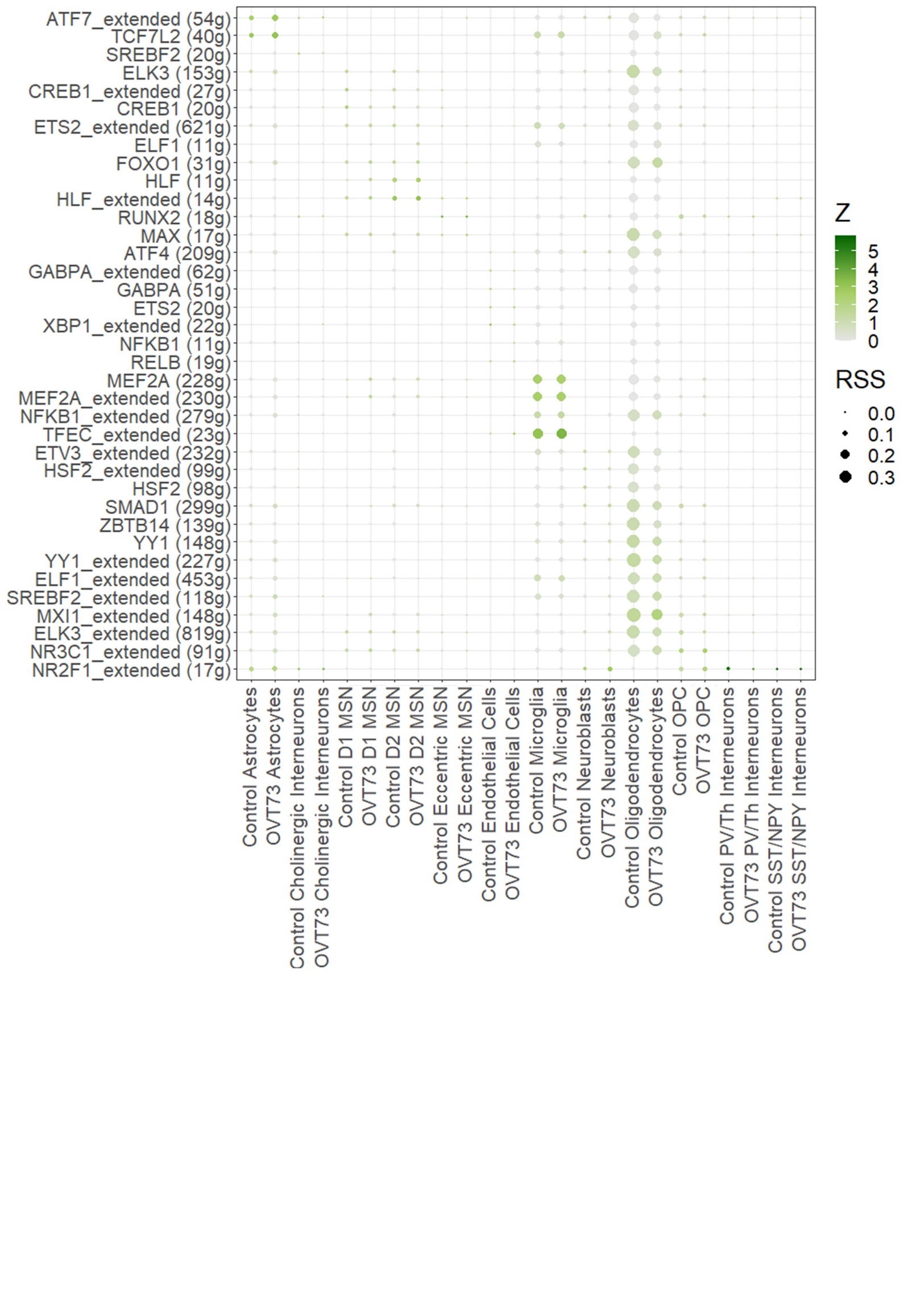


**Supplementary Figure 13 Regulon specificity score of all regulons in the *OVT73* and control cell types.** The regulon specificity score assesses the exclusive regulon activity in cell types. Regulons in cell types with a specificity score of 1 indicates exclusive expression of the regulon in that one cell type, while a specificity score of 0 indicates the regulon is evenly expressed across all cell types. Size of the dot is proportional to the Z score.


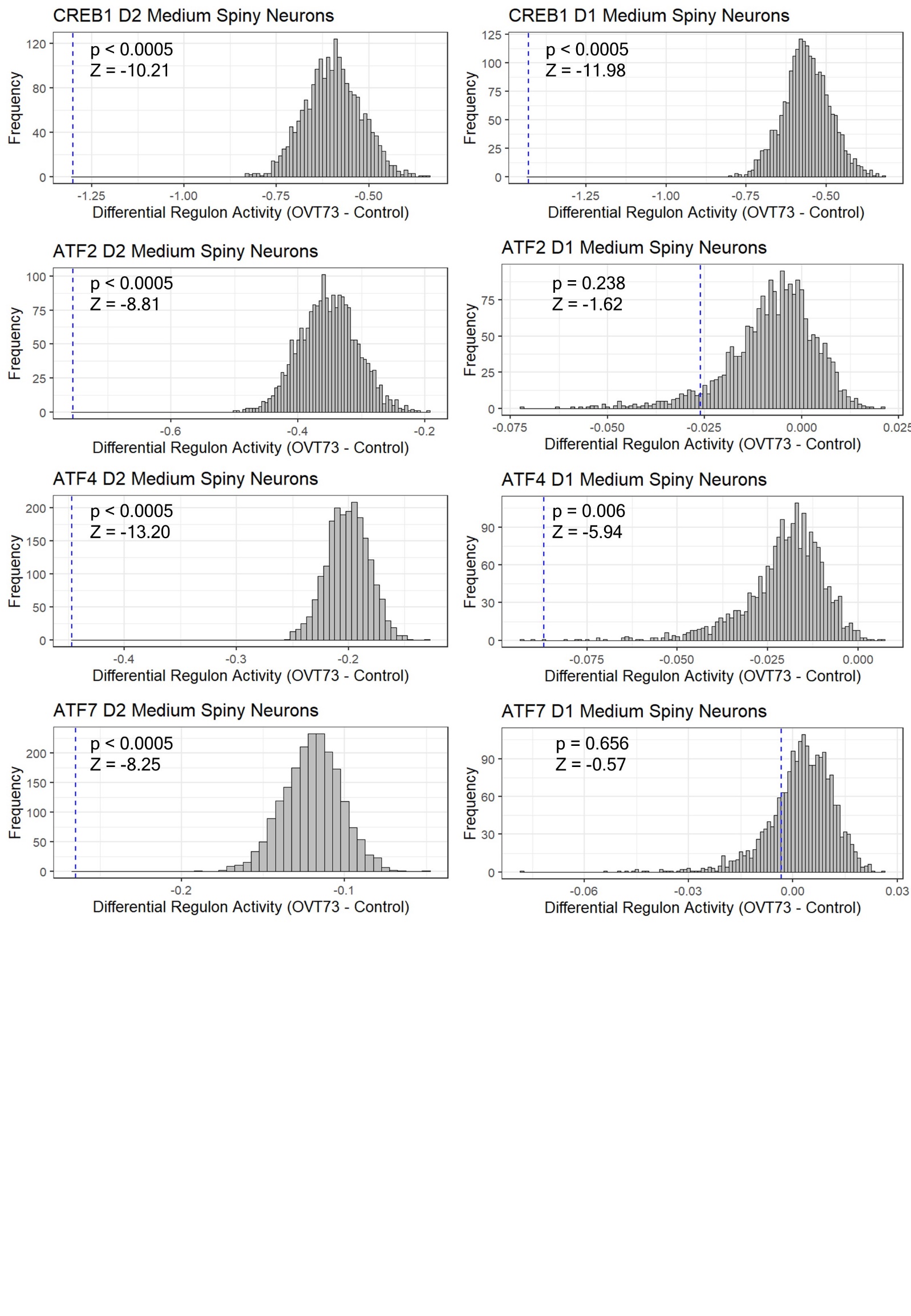


**Supplementary Figure 14** **Distribution of expected differential regulon activity scores between *OVT73* and control when genotype labels are randomly assigned.** Randomised permutation tests were performed with 2,000 permutations. Blue vertical line indicates actual differential regulon activity score.
